# A systems biology approach to find representative genes in Acute Myeloid Leukemia

**DOI:** 10.1371/journal.pone.0352167

**Published:** 2026-07-27

**Authors:** Behnam Aghajan, Mohammad Reza Ghaemi, Ali M. Mosammam, Emran Heshmati, Khosrow Khalifeh

**Affiliations:** 1 Department of mathematics, Faculty of Sciences, University of Zanjan, Zanjan, Iran; 2 Department of statistics, Faculty of Sciences, University of Zanjan, Zanjan, Iran; 3 Department of Biology, Faculty of Sciences, University of Zanjan, Zanjan, Iran; 4 Department of Biotechnology, Research Institute of Modern Biological Techniques, University of Zanjan, Zanjan, Iran; Faculty of Medicine of Tunis, TUNISIA

## Abstract

In this study, we modeled gene expression profile data from Acute Myeloid Leukemia (AML) and healthy cases. At first, the GEO-GSE9476 dataset was processed, and a total of 341 genes were identified as differentially expressed genes (DEGs) in patients, and 599 DEGs in healthy individuals. Gene Ontology and pathway analysis on DEGs led to the identification of 5 Transcription Factors for patients and 3 for healthy cases. Analysis of the respective metabolic pathways revealed a common region in the metabolic pathway between AML and Tuberculosis (TB) that confirmed the validity of our procedure due to the consistency with similar reports. Upon PPI network analysis, Hub genes and three modules containing 41 up-regulated and down-regulated genes in AML patients were identified. Survival analysis on these genes results in reducing the number of identified effective genes into 3 upregulated (*ITGAM*, *ITGAL* and *CD163*) and 5 downregulated genes (*MCM2*, *MCM3*, *RFC4*, *RFC5* and *FEN1*). Finally, drug sensitivity analysis was performed on these genes demonstrating complexity in drug-resistance due to the pattern of gene expression. This knowledge could potentially enable personalized treatment approaches based on individual patient responses due to the epigenetics and life style which affect gene expression pattern.

## 1. Introduction

Acute Myeloid Leukemia (AML) is a type of bone marrow cancer in which a fraction of stem blood cells, instead of transforming into red blood cells, granulocytes, and platelets, transforms into a type of immature white blood cell called a myeloblast. It should be noted that, under normal conditions, bone marrow cells produce blood stem cells, which are biologically considered immature cells. These cells differentiate into myeloid or lymphoid stem cells and eventually mature into red blood cells, granulocytes, and platelets [1–3]. It is clinically and genetically heterogeneous and is classified into six main categories: AML with myelodysplasia-related changes (MRC); therapy-related myeloid neoplasms (t-MN); AML with recurrent genetic abnormalities; myeloid sarcoma; AML, not otherwise specified (NOS); and myeloid proliferations related to Down syndrome [4].

AML as an aggressive form of leukemia demands increased research and development efforts for effective treatment strategies [5,6]. Identifying crucial genetic biomarkers could enhance our understanding of AML initiation and progression. Recurrence and drug resistance pose significant challenges in controlling the disease [7,8]. Various therapeutic methods have been proposed to control and potentially treat the disease [9]. One of the challenges in controlling and treating AML, as well as other chronic diseases, is that current therapeutic strategies are only able to add years to life. However, our main objective must be to increase the quality of life during these years. [10]. In other words, if infectious diseases were the main medical challenge in the first half of the twentieth century, chronic diseases have become the main problem for medicinal biotechnology in modern societies. This is because chronic diseases do not have a single, identifiable cause but rather result from a complex network of interactions between cellular components and environmental factors [11]. Therefore, instead of linearly examining a given disease-causing factor, as it done for infectious diseases [12], it is necessary to investigate the biological networks associated with the occurrence and progression of chronic diseases to identify key components within these networks [13–15]. On the other hand, the occurrence of any trait in living organisms depends on the proper functioning of several proteins. Malfunctioning of one or several proteins, due to excessive or insufficient expression or folding problems, can lead to biological disorders by altering the normal protein network. [16,17]. Accordingly, deducing and comparing protein-based networks, i.e., all expressed proteins in normal and abnormal conditions, can effectively contribute to the development of better strategies for controlling and treating chronic diseases [18].

In the current work, we used a systems biology approach to compare two sets of proteomes from normal and AML-affected cells. After carefully manipulating the raw biological data using appropriate R-package tools and applying statistical restrictions, we constructed and compared differentially expressed networks. We further extended this comparative study to find effective genes in AML.

## 2. Materials and methods

### 2.1. Data sets

In this study, a comprehensive search was performed at the Gene Expression Omnibus database (GEO, ncbi.nlm.nih.gov/geo) using search queries including AML patients and healthy donors.

Gene expression data were obtained from GEO-GSE9476 (last updated Nov 2018), which includes profiles from normal hematopoietic cells (N = 38) and leukemic blasts (N = 26) across various cell types (CD34 + selected cells, N = 18; unselected bone marrow cells, N = 10; unselected peripheral blood cells, N = 10). A GSE dataset contains samples, raw/processed expression files, and experimental metadata. In other words, it represents the full experimental dataset, including all samples and their associated expression values. The dataset was generated using the Affymetrix platform described in GEO-GPL96 (Nov 2007). The GPL record defines the technical platform used to produce the data, including probe design and array specifications. All platform parameters have verified to meet our filtering criteria for probe design and data compatibility. Filtered criteria includes, study type, sample count (at least 20 samples), Homo sapiens, and expression profiling by array. The gene expression profiles were measured with the Affymetrix Human Genome U133A Array platform in Stirewalt Lab at Seattle, USA [19].

### 2.2. Programs

The accession number of the selected dataset begins with the prefix “GSE." It is a matrix with 22,283 rows and 64 columns. Each row, identified by a unique ID, represents a specific probe (corresponding to a specific gene), and each column represents a specific sample. In other words, each row of the matrix represents the gene expression values for individual probes (genes) across 64 samples.

Statistical analysis and data manipulation were performed using appropriate packages in R version 4.2.3. We used Bioconductor packages (Biobase, BiocGenerics, limma, GEOquery) and CRAN packages for graphical representation of images. Protein-Protein Interaction (PPI) network construction and feature deduction were performed using Cytoscape version 3.10.1 with the following apps and plugins: MCODE (version 2.0.3), Mclique (version 1.2), cytoHubba (version 0.1), ModuLand 2.0 (version 3.0.0), and default core applications.

### 2.3. Quality control

Prior to matrix analysis for finding genes and effective metabolic pathways, quality control was performed to remove experimental errors and artifacts that occurred during data acquisition.

Principal component analysis (PCA) was used for quality control of biological observational data. The reason for using this procedure is that the expression matrix (GSE9476) is a 22283-dimensional space, which poses significant challenges for quality control. PCA identified a set of perpendicular axes, known as principal components, that capture the maximum variance of the data. This allows us to reduce the dimensionality of the data and visualize it in two or three dimensions without losing the main information. Upon classification of the data, the possibility of significant gene expression differences between corresponding genes in various samples can be observed [20].

### 2.4. Identification of differentially expressed genes

Upon obtaining the normalized GSE9476 dataset from the GEO database, we identified genes with significant expression levels among the 22,283 probes using two filtering parameters: logFC and P-value. To adjust the P-values and control the false discovery rate (FDR), we employed the Benjamini-Hochberg method, a powerful technique for multiple hypothesis testing. Genes with significant expression levels in healthy and patient cases were identified using the R package and the following criteria:

#### Patients: FDR < 0.02 and logFC > 1 (indicating at least a two-fold increase in expression compared to healthy samples).

Healthy individuals: FDR < 0.02 and logFC < −1 (indicating at least a two-fold decrease in expression compared to patient samples).

### 2.5. Gene ontology and pathway enrichment analysis

Gene ontology and pathway enrichment analysis on DEGs for both healthy and patient groups were performed using the Enrichr, Enrichr-KG and g:profiler. They are web-based tools that allows users to find the basic knowledge about the biological importance of differentially expressed genes via identification of transcription factors and pathways. They also provide a deep analysis accompanied by biological categories regarding the gene list and IDs.

The list of differentially expressed genes (DEGs) identified for healthy and diseased groups was used as input in the Enrichr tool. Transcription factors were selected based on the number of shared genes between the gene sets of each database in Enrichr (where each transcription factor has a gene set based on various databases) and our target genes (i.e., the extracted DEG set). Additionally, the statistical significance of intersections and the repeatability of transcription factors across different databases were considered.

The statistical significance in our transcription factor analysis was determined based on the adjusted p values directly computed by Enrichr. Since enrichment testing in Enrichr involves multiple hypotheses across several databases, the adjusted p value (rather than the raw p value) represents the appropriate metric for assessing significance. In our analysis, only TFs with an adjusted p value < 0.05 were considered statistically meaningful.

Enrichr independently compares the input gene list (341, 599 genes in our cases) with TF–target sets derived from each database. A TF is considered repeatable only if it demonstrates significant enrichment (adjusted p < 0.05) in multiple independent TF based databases.

### 2.6. Functional annotation

The Cytoscape was used to detect and graphical representation of the Gene Ontology (GO). the GEPIA2 tool was used for analysis of gene expression from healthy and cancerous tissues, and that to elucidate the survival analysis [21]. We used BloodSpot as a specialised database for AML patients to get an overview of gene expression patterns of representative genes in healthy and malignant haematopoiesis [22]. The correlation between the expression of the aforementioned up and down-regulated genes in AML patients and the sensitivity of AML cells to small-molecule drugs was investigated by the GDSC IC50 data included in the Gene Set Cancer Analysis (GSCA) database [23].

### 2.7. Overall study design

**Fig 1:**
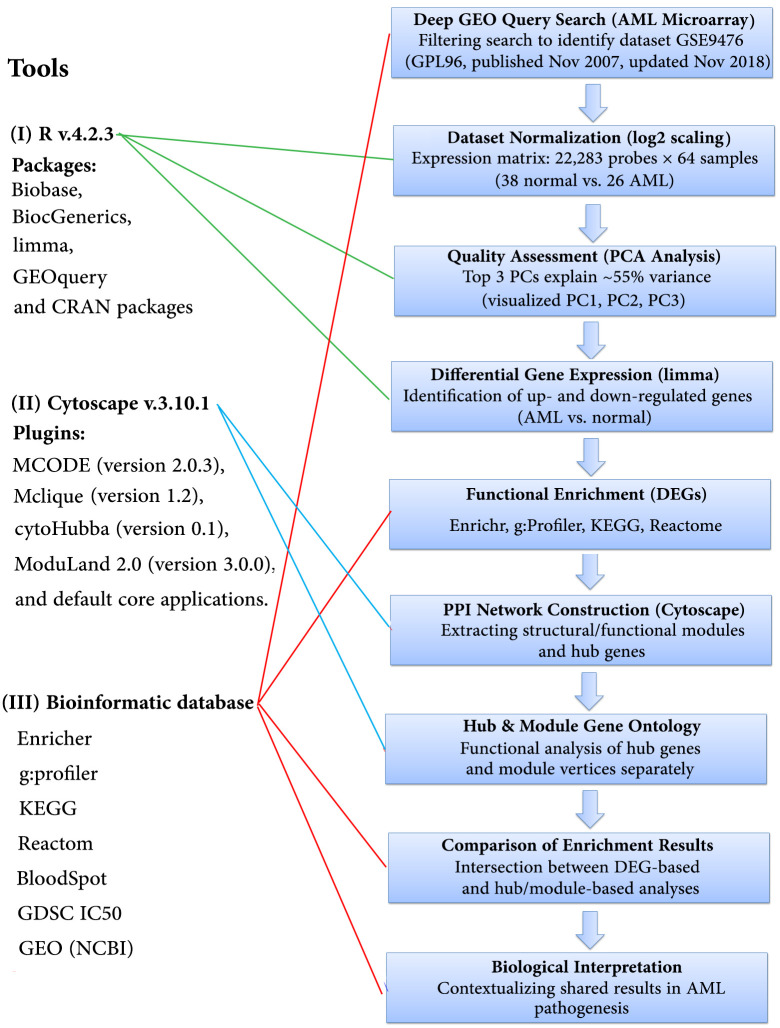
Overall analysis workflow for identifying key genes and pathways in acute myeloid leukemia (AML). This flowchart outlines the nine-step systems biology approach used to analyze gene expression data.

As shown in Fig 1, this study employed a comprehensive, nine-step systems biology workflow to identify key molecular players in acute myeloid leukemia (AML) pathogenesis.

## 3. Results and discussion

### 3.1. Data processing

**Fig 2:**
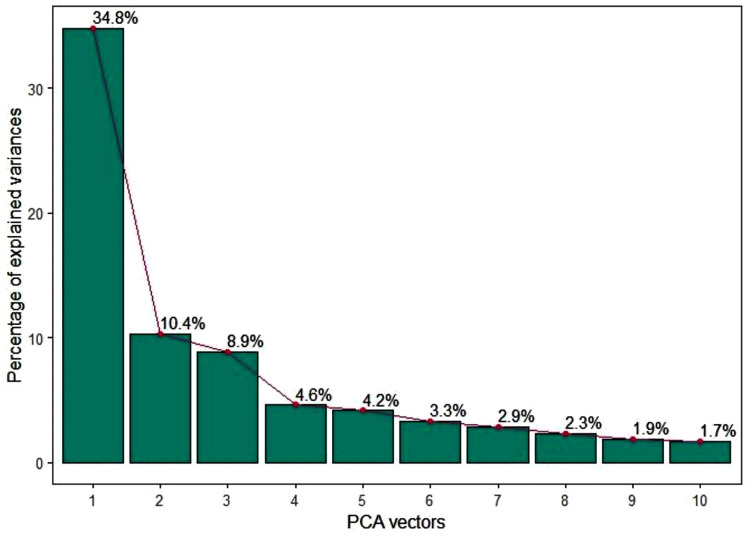
Scree plot of the principal components (eigenvectors) of the expression matrix of the GSE9476 dataset.

Fig 2 presents a Scree Plot of the eigenvalues of the gene expression matrix, ordered from largest to smallest. It depicts the principal components analysis (PCA) relative to gene expression levels in samples, including those genes with significant expression differences.

**Fig 3:**
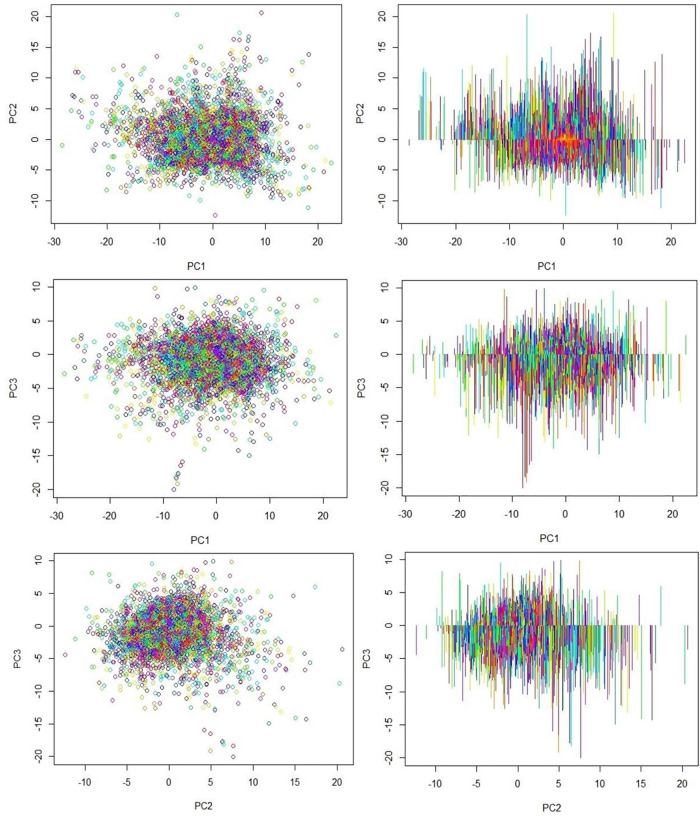
Principal components. The PC1, PC2, and PC3 are provided in pairwise comparisons. The first principal component, PC1, represents the direction along which the data exhibits the greatest variation. The second and third components, PC2 and PC3, correspond to the subsequent directions where the data shows significant variation. Notably, these three components are mutually orthogonal to each other in pairs.

As shown in Fig 2, the genes exhibit a logical distribution. The first three eigenvalues, accounting for approximately 55% of the total variance, were identified as the most significant components. Consequently, PC1, PC2, and PC3 were visualized in Fig 3. PC1 represents the direction of maximum variance in the data. Similarly, PC2 and PC3 capture the second and third highest variances, respectively. Importantly, these components are pairwise orthogonal.

**Fig 4:**
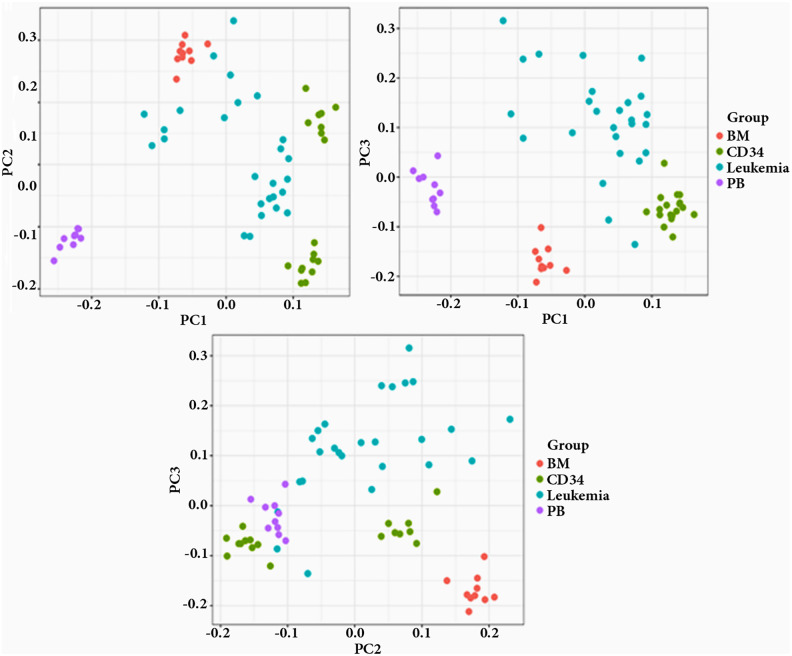
Discrimination between subgroups of healthy and diseased individuals. It illustrates how each of the principal components, PC1, PC2, and PC3, effectively distinguishes between subgroups of healthy and diseased individuals. Specifically, the subgroups PB, BM, CD34, and Leukemia each represent distinct classes. The pairwise comparison of the principal components reveals that the plot of PC1 against PC2 offers enhanced clarity in classification of the data. Furthermore, it suggests that the groups CD34 and Leukemia may contain additional subgroups that have yet to be identified.

Next, the PCA of samples was plotted as shown in Fig 4. It indicates that each group (PB, BM, CD34 and Leukemia) is distinctly classified. This suggests that the data have sufficient quality and reliability for further analysis. The pairwise comparison of PCs reveals that PC1 offers a clearer separation of classes compared to PC2. Additionally, the CD34 and Leukemia subgroups appear to contain further substructures that may should be ignored due to the limitation of the study.

**Fig 5:**
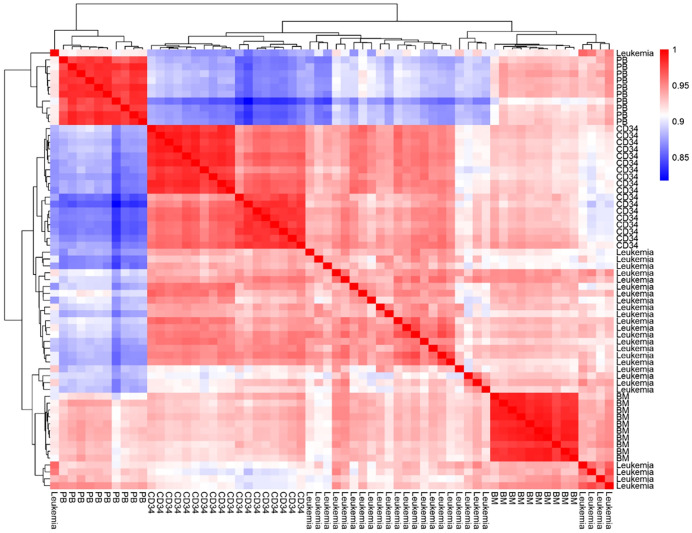
Hierarchical clustering. The correlation between all samples in pairs and their clustering was performed, with the results displayed in the form of a heatmap. By cutting the outermost line of the hierarchical clustering dendrogram, the PB group is separated from the other groups. Additionally, by cutting the next outermost line, a similar separation occurs for the BM group as well.

Fig 5 presents heatmap plots illustrating the pairwise correlation between all samples. Given that cancerous cells often exhibit distinct gene expression profiles, leading to changes in the overall behavior of cells toward maintaining and promotion of cancer, it is surprising to observe that the Leukemia subgroup does not show a significant difference in this regard. To compare the gene expression profiles of healthy individuals and AML patients, we must carefully select appropriate subgroups from BM, PB, CD34, and Leukemia. Based on Fig 5 the PB and BM subgroups lack sufficient similarity and are therefore excluded. Thus, the comparison will focus on the Leukemia and CD34 subgroups.

### 3.2. Differentially expressed genes and transcription factors

Differentially expressed genes (DEGs) represent genes with opposite expression patterns between the two groups. Upon analysis of GEO dataset under specified cut-offs; a total of 341 genes were identified as differentially expressed genes (DEGs) in patients, and 599 genes in healthy individuals. Genes that did not meet the specified criteria were excluded from further analysis.

The list of DEGs from healthy and patient groups was analyzed using Enrichr server, and concerning statistical criteria as mentioned in material and method section. Accordingly, TFs that appeared significantly in at least two independent databases were retained.

The gene list of DEGs was also used in g:Profiler server, and statistically significant TFs were reviewed and confirmed. The results from both tools are provided in Table 1.

**Table 1:** List of Transcription factors upon analysis of DEGs gene list.

Transcription Factor (TF)	Group	Frequency in databases of Enrichr
*SPI1*	AML	6
*IRF8*	AML	4
*NFKB1*	AML	3
*NFE2*	AML	2
*RUNX1*	AML	3
*E2F1*	Healthy	3
*E2F4*	Healthy	3
*GATA1*	Healthy	5

### 3.3. Pathway enrichment analysis

The pathways reported by Enrichr, Enrichr-KG, and g:Profiler were further filtered based on the statistical significance of the results. The results for AML-up and AML-down DEG genes are shown in S1 and S2 Tables, respectively. Interestingly, as can be seen that analysis of the respective metabolic pathways in different metabolome servers with various assumptions, revealed a common region in the metabolic pathway between AML and Tuberculosis (TB). This finding is in good agreement with other reports on the correlation between AML and TB [24–26], and can be considered as a reliability test of our procedure.

### 3.4. PPI network analysis

**Fig 6:**
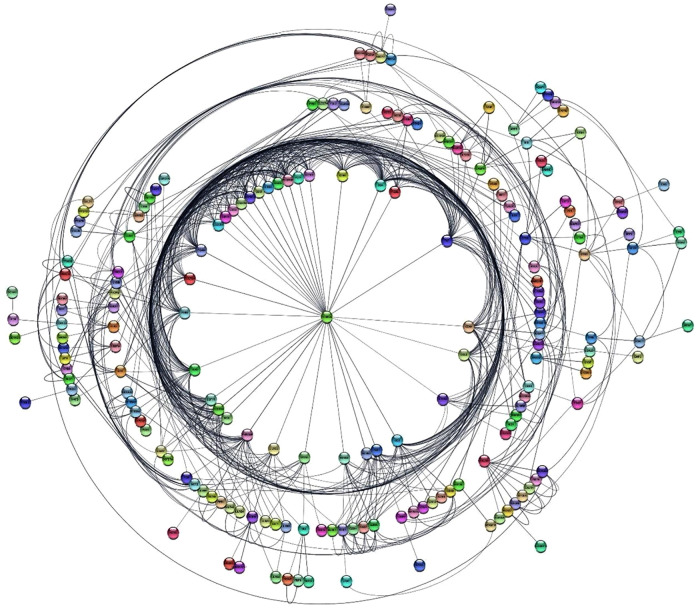
The PPI network for the AML-UP group. PPI network was organized using a radial layout algorithm. It positions the nodes (vertices) on virtual concentric circles around a common center.

**Fig 7:**
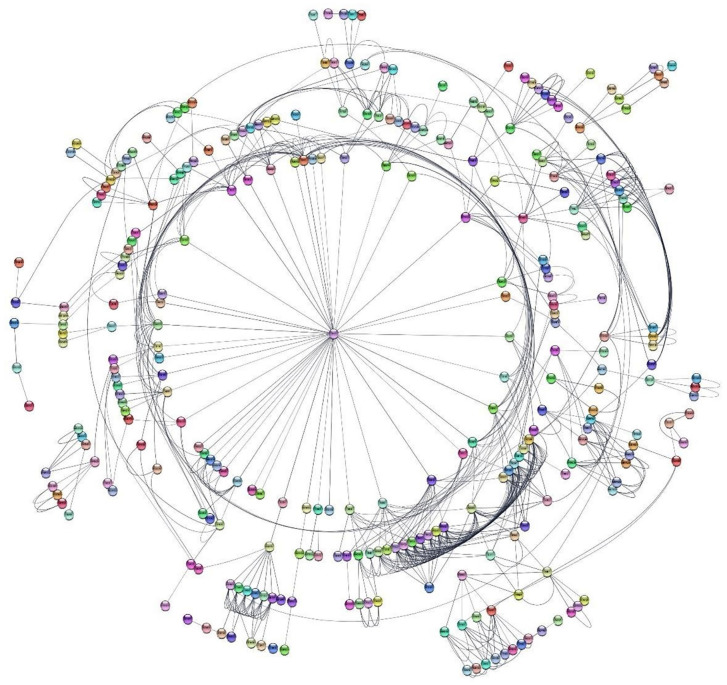
The PPI network for the AML-down group. PPI network was organized using a radial layout algorithm. It positions the nodes (vertices) on virtual concentric circles around a common center.

By using yFiles Layout Algorithms app a total of 340 identifiers were identified for AML-up and 607 identifiers for AML-down. The first one formed a protein-protein interaction (PPI) network with 340 nodes and 668 edges, while the corresponding network for the AML-down group contained 607 nodes and 718 edges. Since these networks did not consist solely of isolated nodes and contained many short paths (lengths 2 and 3) with minimal impact on network, these nodes were ignored from the corresponding networks (Table 2). The maximum connected subgraphs were then used for further analysis of the AML and healthy networks, as shown in Figs 6 and 7.

**Table 2:** An analysis of the characteristics of the core networks for the AMLup and AMLdown groups was conducted, focusing on their maximum connected subnetworks. As indicated in the table, the two groups differ only in the number of nodes, edges, and connected components, while they do not exhibit significant differences in other important network features. The metrics presented in this table were calculated using NetworkAnalyzer app (v4.4.8).

Summary statistics	Network of Healthy group	Max.con.subNet of Healthy group	Network of AML group	Max.con.subNet of AML group
Number of nodes	**607**	**287**	***340***	***186***
Number of edges	**718**	**688**	***668***	***644***
Average number of neighbors	4.794	4.794	6.925	6.925
Network diameter	14	14	8	8
Network radios	8	8	5	5
Characteristics path length	5.116	5.116	3.379	3.379
Clustering coefficient	0.354	0.354	0.325	0.325
Network density	0.017	0.017	0.037	0.037
Network heterogeneity	1.013	1.013	1.126	1.126
Network centralization	0.131	0.131	0.170	0.170
Connected component	**291**	**1**	***135***	***1***

#### 3.4.1. Hub genes and significant modules of the PPI network.

The definition of a “hub” in a static biological network can be multifaceted. Our approach in this manuscript defines hubs based on a combination of structural and functional characteristics

Specifically, we considered nodes as hubs if they exhibit high centrality measures (e.g., degree centrality, betweenness centrality), indicating the structural importance and potential of a node to control information flow or connect other nodes within the network. Also, the hubs should be located within key functional modules, where they reside in clusters of nodes that perform related biological functions. Hence, nodes exhibiting high centrality measures within the static biological network, and simultaneously residing in key functional modules, are defined as “hubs.”

**Fig 8:**
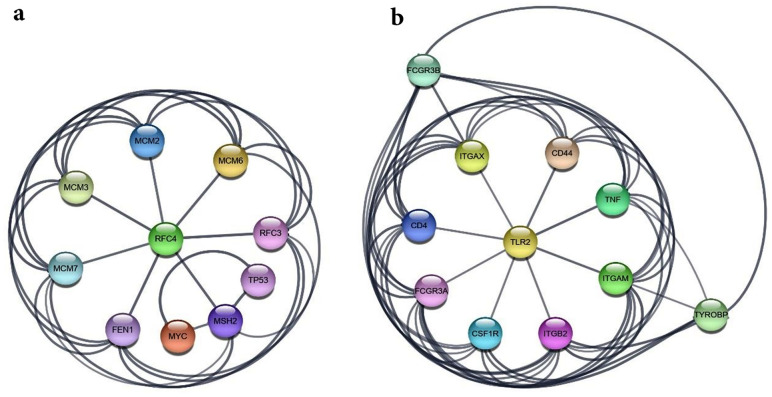
Neighborhood of the hub genes with each other. **(a)** Neighborhood of 10 hub genes of the AML-up network and **(b)** neighborhood of 11 hub genes of the AML-down network.

Table 3 lists the hub genes obtained from the analysis of the networks shown in Figs 6 and 7. This table presents the degree ranking as well as various centrality and closeness metrics for each hub gene. Hub genes (genes with high potential for being biologically significant hubs) were selected based on structural and functional network criteria. Specifically, for the AML-up network, the top 11 genes, and for the AML-down network, the top 10 genes with higher degree, betweenness centrality, and closeness centrality compared to other nodes were identified as hubs. These criteria were evaluated separately for each network, considering their different density and clustering properties. As shown in Fig 8, the hub genes from both networks form a connected subgraph due to their specific interactions.

**Table 3:** The hub genes for the two networks, AMLup (n = 11) and AMLdown (n = 10), are presented along with their degree sequences and several network statistical metrics for each node. The evaluation of these metrics was calculated to four decimal places.

Hub Genes	Network	Degree	Clustering coefficient	Closeness centrality	Betweenness centrality	Neighborhood connectivity
*CD4*	AMLup	38	0.2745	0.4373	0.0741	16.2894
*TNF*	AMLup	36	0.2079	0.4579	0.1835	15.0833
*ITGAM*	AMLup	33	0.3693	0.4292	0.0486	18.5757
*TLR2*	AMLup	32	0.2620	0.4233	0.0765	15.7812
*FCGR3A*	AMLup	32	0.4133	0.4233	0.0264	19.75
*TYROBP*	AMLup	31	0.2150	0.4195	0.1223	14.3548
*ITGB2*	AMLup	30	0.3310	0.4383	0.0715	18.8333
*FCGR3B*	AMLup	27	0.4159	0.4129	0.0263	19.5185
*CSF1R*	AMLup	26	0.3846	0.4185	0.0454	19.2692
*CD44*	AMLup	25	0.26	0.4092	0.1076	16.08
*ITGAX*	AMLup	25	0.4667	0.4065	0.0210	19.84
*TP53*	AMLdown	42	0.0673	0.3268	0.3964	6.8809
*MYC*	AMLdown	23	0.1501	0.3058	0.1181	8.8695
*MCM6*	AMLdown	21	0.4095	0.2690	0.0963	12.7142
*FEN1*	AMLdown	20	0.4947	0.2614	0.0191	13.85
*MCM3*	AMLdown	20	0.4789	0.2640	0.0344	13.5
*RFC4*	AMLdown	20	0.5368	0.2454	0.0053	13.75
*MCM2*	AMLdown	18	0.5163	0.2602	0.0142	13.3334
*MCM7*	AMLdown	18	0.5098	0.2569	0.0137	13.6667
*MSH2*	AMLdown	17	0.3823	0.2820	0.0416	15.2941
*RFC3*	AMLdown	17	0.6176	0.2489	0.0066	14.2352

**Fig 9:**
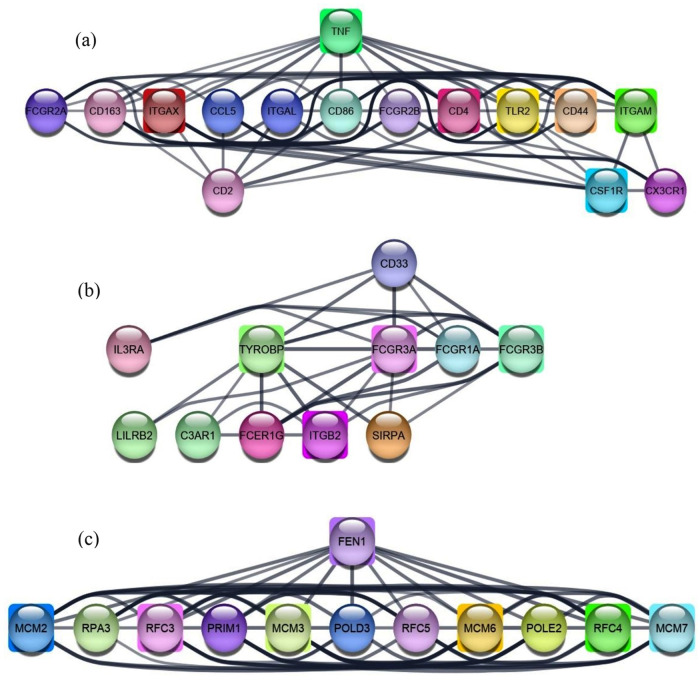
Network analysis. (a) and (b) show the modules of the AML-up network, while (c) illustrates the module of the AML-down network. The identified hub genes listed in Table 3 for both networks are highlighted in each module with a colored square behind each gene. In module (a), seven hub genes are identified, while module (b) contains four hub genes, and module (c) also identifies seven hub genes from those listed in Table 3.

Examination of network modules for the AML-up and AML-down networks revealed that each of them contains several gene hubs (Fig 9).

Modules of both AML-up and AML-down networks then were identified using plugin MCODE. By examining these modules, it was revealed that each module contains several gene hubs which provided in Table 3, as can be seen in Fig 9.

Table 4 shows the top 12 genes that meet the most criteria including clustering coefficient (Clu.co), closeness centrality (Clo.ce), betweenness centrality (Bet.ce), neighborhood connectivity (Nei.con), and MCODE score for both the AML-up and AML-down groups. We then compared the gene hubs from both networks to identify common genes, which are likely to be the most important genes for further investigation.

**Table 4:** A list of genes (the top 24 genes) that exhibit the highest values for the metrics of clustering coefficient (Clu.co), closeness centrality (Clo.ce), betweenness centrality (Bet.ce), neighborhood connectivity (Nei.con), and MCODE score.

gene	Clu.Co	gene	Clo.ce	gene	Bet. ce	gene	Nei. con	gene	MCODE score
AML UP PPI Network									
*IL17RA*	1.0	**TNF**	0.4579	**TNF**	0.1835	IL17RA	37.0	ITGAL	9.0
*VCAN*	1.0	**ITGB2**	0.4383	**TYROBP**	0.1223	BAG4	36.0	FCGR2A	8.8363
*LILRA2*	1.0	**CD4**	0.4373	**CD44**	0.1076	IRAK3	32.0	FCGR2B	8.8363
*GFI1*	1.0	**ITGAM**	0.4292	CTSS	0.0895	CLEC4A	32.0	**ITGAX**	8.6666
*CYBA*	1.0	**FCGR3A**	0.4233	CALM3	0.0876	LST1	31.0	**CD44**	8.0769
*KLRF1*	1.0	**TLR2**	0.4233	**TLR2**	0.0765	CORO1A	30.0	CX3CR1	8.0
*CTSA*	1.0	**TYROBP**	0.4195	**CD4**	0.0741	LCP1	30.0	CD2	8.0
*SH2D1A*	1.0	**CSF1R**	0.4185	**ITGB2**	0.0715	VCAN	28.5	**CSF1R**	7.9615
*IL13RA1*	1.0	**FCGR3B**	0.4129	IQGAP1	0.0691	FUT4	27.3334	**TLR2**	7.9120
*NAIP*	1.0	**CD44**	0.4092	FLT3	0.0551	ITGAL	26.25	CD86	7.7778
*WT1*	1.0	**ITGAX**	0.4065	CTSD	0.0519	IL3RA	26.0	**TNF**	7.6285
*ADD3*	1.0	CCL5	0.4065	**ITGAM**	0.0486	CSF2RA	26.0	**CD4**	7.5438
AML Down PPI Network									
*ELOVL6*	1.0	**TP53**	0.3268	**TP53**	0.3964	BTG2	42.0	RPA3	9.0
*TRMT11*	1.0	**MYC**	0.3058	**MYC**	0.1181	ZBTB16	42.0	**MCM7**	9.0
*GSTM2*	1.0	JUN	0.2969	IL1B	0.1105	CCNG1	42.0	RFC5	8.8363
*PKIA*	1.0	PARP1	0.2942	**MCM6**	0.0963	OBSL1	42.0	PRIM1	8.8363
*GCSH*	1.0	CDK4	0.2942	RUVBL2	0.0955	PHGDH	32.5	POLE2	8.8363
*BAG2*	1.0	CASP3	0.2840	DDX10	0.0856	NDN	25.5	**RFC3**	8.8363
*GSTM1*	1.0	SMAD4	0.2837	CTPS1	0.0814	AKT3	24.3334	**FEN1**	8.8363
*MZT2B*	1.0	MSH6	0.2834	JUN	0.0799	SOX4	23.0	**RFC4**	8.8363
*HACD1*	1.0	IL1B	0.2831	NME7	0.0791	METTL3	23.0	**MCM3**	8.8363
*FANCL*	1.0	**MSH2**	0.2820	SNRPN	0.0705	G3BP1	22.3334	**MCM2**	8.8363
*MZT2A*	1.0	CCT4	0.2776	NDN	0.0705	KEAP1	22.0	**MCM6**	8.8363
*NUP133*	1.0	CDH1	0.2757	SMAD4	0.0690	CSNK2A	22.0	POLD3	8.0

#### 3.4.2. Biological analysis of Hub Genes and significant modules.

**Fig 10:**
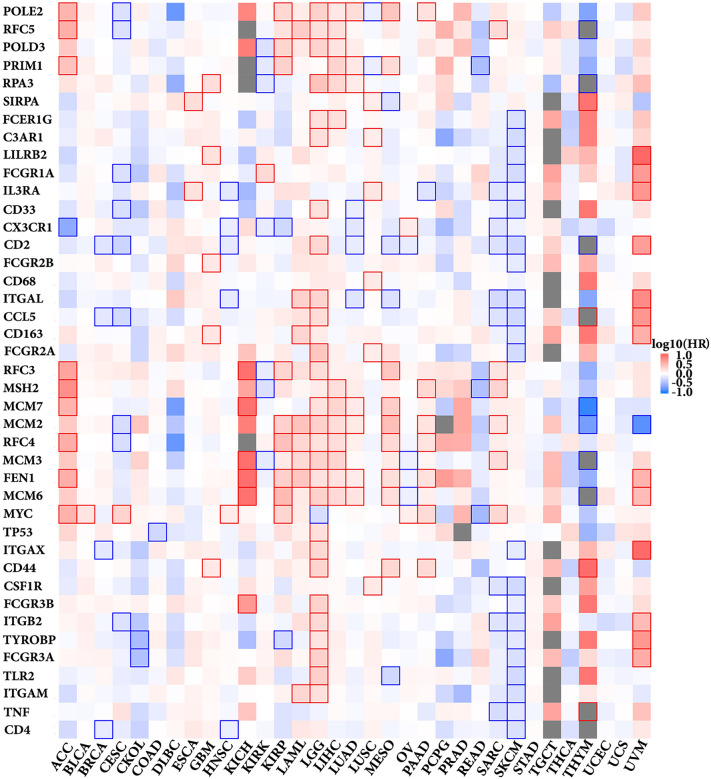
Comprehensive Survival Heatmap – A rigorous GEPIA2 analysis presenting log10(Hazard Ratio) for 41 hub and module genes across several cancer types (33 cases) derived from TCGA datasets. The heatmap, constructed using overall survival metrics with a median cutoff, 0.05 significance level (no p-value adjustment), and monthly time units, employs a color gradient where red indicates adverse prognosis (log10(HR) > 0), blue denotes favorable prognosis (log10(HR) < 0), gray signifies unavailable data, and white represents neutral effects (log10(HR) ≈ 0). Highlighting eight genes with consistent weak adverse signals in AML, this visualization elucidates potential mechanistic overlaps with related malignancies, offering critical insights into prognostic biomarkers and therapeutic targets for AML research.

Our mathematical analysis identified Gene hubs and three representative modules within Fig 9, encompassing 41 genes derived from differentially expressed genes (DEGs). Network analysis revealed these genes exhibit significant control within protein interaction networks. To further investigate these genes, we utilized GEPIA2 to assess gene expression in healthy and cancerous tissues and conduct survival analysis. This analysis elucidated the correlation between gene expression levels and patient survival across various cancer types. The survival heatmap in Fig 10 illustrates the overall survival of these genes from diagnosis or treatment initiation to death from any cause. The heatmap graphically depicts the prognostic significance (higher risk of a negative or positive event) of these genes in different cancers, with color variations indicating higher or lower gene expression levels correlated with the risk of an event compared to the control group. In AML, our analysis revealed that upregulation of three genes (*ITGAM*, *ITGAL*, and *CD163*) and downregulation of five genes (*MCM2*, *MCM3*, *RFC4*, *RFC5*, and *FEN1*) are associated with higher risk of a negative event, suggesting high hazard ratios (HR) for AML.

The comparison between the up- and downregulated genes in AML and a group of healthy individuals (P < 0.05) and overall survival (OS) from GEPIA2 are provided in S3 and S4 Figs, respectively. The results demonstrate the pattern of the 8 candidate genes that are important as prognostic signal for AML disease [27].

Additionally, we employed the BloodSpot tool to determine the expression levels of these genes in AML patients. Notably, all five high-expressed genes exhibited similar expression patterns in specific cell types. This suggests that these genes may play similar roles in AML pathogenesis, potentially contributing to abnormal blood cell production and the manifestation of various clinical symptoms, such as anemia, increased infection risk, and blood clotting problems [28].

**Fig 11:**
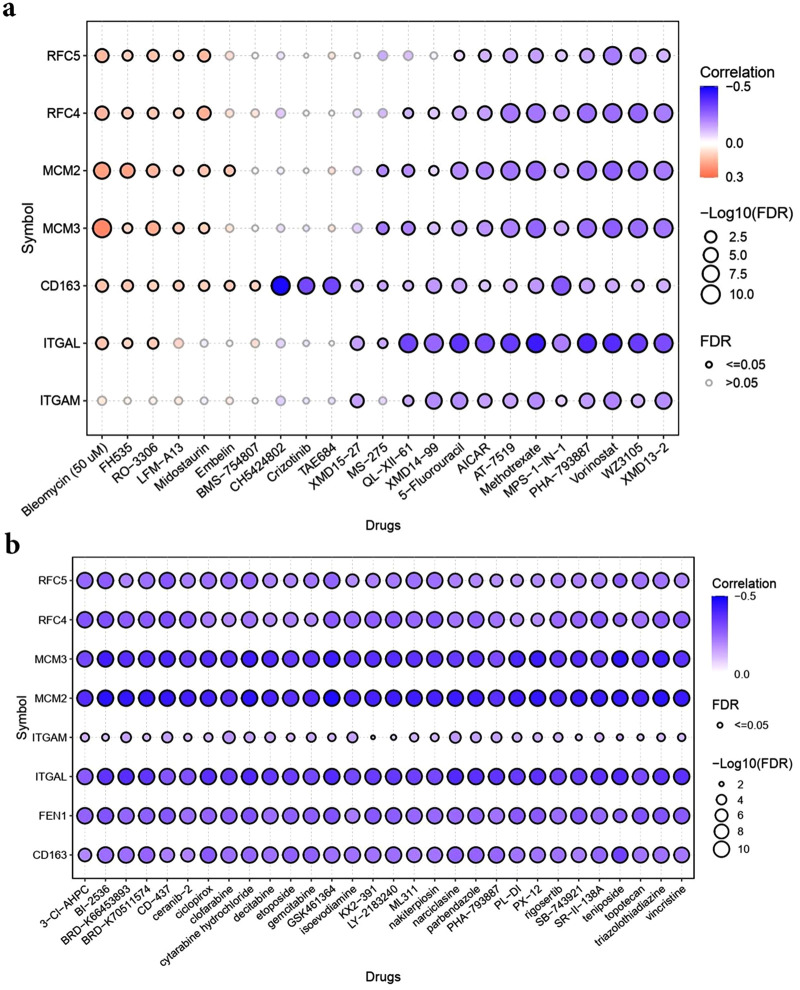
Sensitivity analysis. The Figure summarizes the correlation between expression of representative genes (*RFC5*, *RFC4*, *MCM3*, *MCM2*, *CD163*, *ITGAL*, *ITGAM*) and the sensitivity of (a) top 30 GDSC drugs and (b) top 30 CTRP drugs. The sensitivity is measured using IC50 values, representing the concentration of drug required to inhibit cell growth by 50%. Based on the database criteria and an FDR of less than 0.05, the *FEN1* gene did not show significant resistance or sensitivity to any of the analyzed drugs in GDSC database.

Drug sensitivity assessment was conducted using data from the Genomics of Drug Sensitivity in Cancer (GDSC) and Cancer Therapeutics Response Portal (CTRP) *via* Pan-cancer analysis, with results presented in Fig 11a, b, respectively.

The respective algorithm uses Pan-cancer analysis which involves assessing frequently mutated genes and other genomic abnormalities common to many different cancers, regardless of tumor origin. Each point in these diagrams represents a specific drug on the x-axis and a given gene on the y-axis. Higher values of -Log10(FDR) indicate statistically more significant correlations. A positive correlation in Fig 11a suggests that higher gene expression is associated with drug resistance. For instance, *MCM2* and *MCM3* exhibited the highest positive correlation with Bleomycin (50uM), indicating increased resistance to this drug upon increasing the expression level. Accordingly, their low expression in AML patients (as down-regulated genes for AML patients) is associated with increased drug sensitivity, indicating need for lower concentration of Bleomycin(50uM) for treatment. Additionally, *CD163* shows significant negative correlation with CH5424802 demonstrating that its high expression is associated with sensitivity to this drug. Similarly, high expression of *ITGAL* is accompanied by more sensitivity to Methotrexate.

In summary, both *MCM2* and *MCM3*genes were confirmed to be down‑regulated in AML patients when compared with healthy controls. Their expression shows a positive correlation with Bleomycin resistance (Fig 11a). However, their elevated expression showed negative correlation with sensitivity scores across several CTRP drugs (Fig 11b). This seemingly opposite pattern reflects the drug‑specific and context‑dependent nature of these genes in chemotherapeutic response. Low expression of MCM2 and MCM3 in AML may thus enhance sensitivity to certain agents (e.g., Bleomycin) while reducing responsiveness to others, underscoring the complexity of therapeutic targeting in AML.

Fig 11b revealed that *MCM2*, *MCM3* (down-regulated in AML patients), and *ITGAL* (up-regulated in AML patients) exhibited the most significant negative correlations with the top 30 drugs, suggesting that higher expression of these genes leads to increased sensitivity to the top 30 drugs. Resistance of *MCM2* and *MCM3* to Bleomycin(50uM) (Fig 11a) and sensitivity to GDSC drugs (Fig 11b) suggests that high expression of these genes is associated with both drug resistance and sensitivity, highlighting the complexity of drug response in AML treatment.

One possible explanation for observing complex behavior against various drugs is the presence of compensatory mechanisms. Lower or higher expression of the representative genes in AML patients as suggested in our model, may be accompanied by the development of alternative adaptive signaling pathways, leading to drug resistance/sensitivity. The tumor microenvironment may also significantly influence the relationship between gene expression and drug sensitivity. Factors such as hypoxia, nutrient availability, and interactions with stromal cells can potentially alter the function of these genes in AML tissues compared to normal ones. Furthermore, these genes may play a protective role against drug toxicity in normal conditions. However, in the context of AML, their reduced/increased expression may not provide the same protective effects due to oncogenic alterations within the tissue. Finally, influencing the gene expression pattern from the environmental conditions highlights the possibility of epigenetics and life style in response to treatment.

## 4. Conclusion

Chronic disease originated from a complex network of interactions between cellular elements and environmental factors. In other words, at the bottom of biological processes, gene expression profile is affected by environmental factors under the concept of epi-genetics. Due to this complexity, diagnosis, control and treatment of chronic diseases is a key challenge in modern medicinal biotechnology. However, the availability of a huge amount of gene expression data for various biological states, and thanks to the capability of advanced algorithms, it is possible to construct meaningful models to identify the key elements of chronic diseases. Here, we have tried to reduce the complexity associated with the AML, by modeling the experimentally determined gene expression profile from AML patients and healthy cases. Finally, 3 upregulated and 5 downregulated genes were identified which their expression pattern is specific for AML disease. Identification of these genes raise a question regarding their response to selected drugs. Bioinformatics analysis on the representative genes in AML-affected tissues revealed that expression patterns of these genes in individual patients determine their responses to drug. Among the identified genes, the increased expression of the three upregulated genes is associated with a worse prognosis in AML patients, suggesting their potential role in disease progression. This finding underscores the importance of precisely understanding gene expression patterns for predicting clinical outcomes. Due to the identity of the *in silico studies*, further experimental validation to complement and extend the findings of this work is required. For example, to confirm the differential expression and biological relevance of reported TFs, the mRNA and protein levels of these TFs must be experimentally validated via direct comparison between primary AML patient samples and healthy donor samples using appropriate moilecular biology approaches. Additionally, to demonstrate a causal role for these TFs in mediating drug resistance or sensitivity, performing *in vitro* and/or *in vivo* assays including knockdown/overexpression followed by drug sensitivity testing in AML cell lines or primary cells is suggested.

## Supporting information

S1 Table The list of identified biological processes for the AMLup group. (DOCX)

S2 Table The list of identified biological processes for the AMLdown group. (DOCX)

S3 Fig The expression levels of the eight candidate genes in AML. The results are based on TCGA and GTEx data screening in AML (n = 173) and normal control (n = 70) from GEPIA2 database through the Expression DIY (box plot) (*, P < 0.05), the red boxes are represented as the AML sample and the black boxes are represented as the normal tissue. CD163; CD163 molecule, FEN1; flap structure-specific endonuclease 1, ITGAL; integrin subunit alpha L, ITGAM; integrin subunit alpha M, MCM2; minichromosome maintenance complex component 2, MCM3; minichromosome maintenance complex component 3, RFC4; replication factor C subunit 4, RFC5; replication factor C subunit 5, AML, acute myeloid leukemia; TCGA, The Cancer Genome Atlas, GTEx project; Genotype-Tissue Expression.
(TIFF)

S4 Fig Kaplan-Meier survival curves of overall survival for the eight candidate genes in AML. The survival curves are plotted using the GEPIA2 web server. Survival curves are represented as dotted lines, and the solid line represents the 95% confidence interval. The number of AML and normal bone marrow tissues (n) =53. The P values are calculated using log-rank statistics. CD163; CD163 molecule, FEN1; flap structure-specific endonuclease 1, ITGAL; integrin subunit alpha L, ITGAM; integrin subunit alpha M, MCM2; minichromosome maintenance complex component 2, MCM3; minichromosome maintenance complex component 3, RFC4; replication factor C subunit 4, RFC5; replication factor C subunit 5, AML, acute myeloid leukemia; HR, hazard ratio.
(TIFF)
