# Supplementary material for: A systems biology approach to find representative genes in Acute Myeloid Leukemia
Source: PLoS One. 2026 Jul 27;21(7):e0352167. doi: 10.1371/journal.pone.0352167 (PMC13405072; doi:10.1371/journal.pone.0352167)
Supplement: S1 Table — (DOCX) [file pone.0352167.s001.docx]

| Database | FDR/q-Value | ID | Term |
| --- | --- | --- | --- |
| Enrichr | | | |
| [Reactome 2022](https://maayanlab.cloud/Enrichr/enrich) | 3.348×10^-47^ | R-HSA-168256 | Immune System |
| Reactome 2022 | 2.946×10^-40^ | R-HSA-168249 | Innate Immune System |
| Reactome 2022 | 3.038×10^-39^ | R-HSA-6798695 | Neutrophil Degranulation |
| Reactome 2022 | 4.168×10^-17^ | R-HSA-198933 | Immunoregulatory Interactions Between a Lymphoid and A non-Lymphoid Cell |
| WikiPathway 2023 Human | 4.126×10^-12^ | WP3945 | TYROBP Causal Network in Microglia |
| WikiPathway 2023 Human | 3.124×10^-11^ | WP3937 | Microglia Pathogen Phagocytosis Pathway |
| KEEG | 5.299×10^-17^ | ko05152 | **Tuberculosis** |
| KEEG | 7.915×10^-16^ | [ko04380](https://www.genome.jp/entry/ko04380) | Osteoclast differentiation |
| [GO Biological Process 2023](https://maayanlab.cloud/Enrichr/enrich) | 1.774×10^-10^ | GO:0032103 | Positive Regulation of Response to External Stimulus |
| [GO Biological Process 2023](https://maayanlab.cloud/Enrichr/enrich) | 1.118×10^-9^ | GO:0031349 | Positive Regulation of Defense Response |
| [GO Cellular Component 2023](https://maayanlab.cloud/Enrichr/enrich) | 3.927×10^-22^ | GO:0030667 | Secretory Granule Membrane |
| [GO Cellular Component 2023](https://maayanlab.cloud/Enrichr/enrich) | 4.147×10^-14^ | GO:0101002 | Ficolin-1-Rich Granule |
| [GO Molecular Function 2023](https://maayanlab.cloud/Enrichr/enrich) | 2.685×10^-6^ | GO:0004896 | Cytokine Receptor Activity |
| [GO Molecular Function 2023](https://maayanlab.cloud/Enrichr/enrich) | 2.685×10^-6^ | GO:0032393 | MHC Class I Receptor Activity |
| Enrichr-KG | | | |
| Go Biological Process 2021 | 5.951×10^-41^ | GO:0043312 | Neutrophil Degranulation |
| KEEG | 5.299×10^-17^ | ko05152 | **Tuberculosis** |
| KEEG | 7.915×10^-16^ | [ko04380](https://www.genome.jp/entry/ko04380) | Osteoclast differentiation |
| Go Biological Process 2021 | 9.0559×10^-17^ | GO:0050776 | regulation of immune response |
| g: profiler | | | |
| KEEG | 4.04×10^-13^ | ko05152 | **Tuberculosis** |
| KEEG | 3.645×10^-12^ | [ko04380](https://www.genome.jp/entry/ko04380) | Osteoclast differentiation |
| Reactome 2022 | 4.459×10^-38^ | R-HSA-168256 | Immune System |
| Reactome 2022 | 1.258×10^-35^ | R-HSA-6798695 | Neutrophil Degranulation |
| Reactome 2022 | 3.28×10^-33^ | RHSA-168249 | Innate Immune System |
| Reactome 2022 | 1.587×10^-10^ | R-HSA-198933 | Immunoregulatory Interactions Between a Lymphoid and A non-Lymphoid Cell |
| WikiPathway 2023 Human | 4.414×10^-10^ | WP3945 | TYROBP Causal Network in Microglia |
| WikiPathway 2023 Human | 1.927×10^-9^ | WP3937 | Microglia Pathogen Phagocytosis Pathway |
| [GO: BP](https://maayanlab.cloud/Enrichr/enrich) | 1.611×10^-51^ | [GO:0002376](https://biit.cs.ut.ee/gprofiler/convert?organism=hsapiens&query=GO:0002376) | immune system process |
| [GO: BP](https://maayanlab.cloud/Enrichr/enrich) | 2.634×10^-44^ | [GO:0006955](https://biit.cs.ut.ee/gprofiler/convert?organism=hsapiens&query=GO:0006955) | immune response |
| GO: CC | 5.214×10^-41^ | [GO:0031982](https://biit.cs.ut.ee/gprofiler/convert?organism=hsapiens&query=GO:0031982) | vesicle |
| GO: CC | 3.927×10^-40^ | [GO:0030141](https://biit.cs.ut.ee/gprofiler/convert?organism=hsapiens&query=GO:0030141) | secretory granule |
| GO: MF | 2.693×10^-17^ | [GO:0140375](https://biit.cs.ut.ee/gprofiler/convert?organism=hsapiens&query=GO:0140375) | immune receptor activity |
| GO: MF | 1.036×10^-16^ | [GO:0019899](https://biit.cs.ut.ee/gprofiler/convert?organism=hsapiens&query=GO:0019899) | enzyme binding |
