# Supplementary material for: A systems biology approach to find representative genes in Acute Myeloid Leukemia
Source: PLoS One. 2026 Jul 27;21(7):e0352167. doi: 10.1371/journal.pone.0352167 (PMC13405072; doi:10.1371/journal.pone.0352167)
Supplement: S2 Table — (DOCX) [file pone.0352167.s002.docx]

| Database | FDR/q-Value | ID | Term |
| --- | --- | --- | --- |
| Enrichr | | | |
| [Reactome 2022](https://maayanlab.cloud/Enrichr/enrich) | 4.039×10^-7^ | R-HSA-1640170 | Cell Cycle |
| [Reactome 2022](https://maayanlab.cloud/Enrichr/enrich) | 5.334×10^-7^ | R-HSA-69190 | DNA Strand Elongation |
| Reactome 2022 | 7.802×10^-6^ | R-HSA-69278 | Cell Cycle, Mitotic |
| Reactome 2022 | 5.51×10^-5^ | R-HSA-69306 | DNA Replication |
| WikiPathway 2023 Human | 5.414×10^-9^ | WP466 | DNA Replication |
| WikiPathway 2023 Human | 2.021×10^-3^ | WP179 | Cell Cycle |
| KEEG | 9.340×10^-9^ | ko03030 | DNA replication |
| KEEG | 3.599×10^-3^ | ko04110 | Cell cycle |
| [BioPlanet 2019](https://maayanlab.cloud/Enrichr/enrich) | 3.294×10^-9^ | - | DNA strand elongation |
| [BioPlanet 2019](https://maayanlab.cloud/Enrichr/enrich) | 1.422×10^-8^ | - | Cell cycle |
| [BioPlanet 2019](https://maayanlab.cloud/Enrichr/enrich) | 1.964×10^-6^ | - | DNA replication |
| [GO Biological Process 2023](https://maayanlab.cloud/Enrichr/enrich) | 4.623×10^-7^ | GO:0006259 | DNA Metabolic Process |
| [GO Biological Process 2023](https://maayanlab.cloud/Enrichr/enrich) | 4.623×10^-7^ | GO:0006261 | DNA-templated DNA Replication |
| GO Cellular Component 2023 | 2.777×10^-7^ | GO:0005634 | Nucleus |
| GO Cellular Component 2023 | 1.405×10^-6^ | GO:0043231 | Intracellular Membrane-Bounded Organelle |
| GO Molecular Function 2023 | 1.723×10^-3^ | GO:0017116 | Single-Stranded DNA Helicase Activity |
| GO Molecular Function 2023 | 1.245×10^-2^ | GO:0003677 | DNA Binding |
| Enrichr-KG | | | |
| KEEG | 9.3397×10^-9^ | ko03030 | DNA replication |
| Go Biological Process 2021 | 3.272×10^-7^ | GO:0006261 | DNA-dependent DNA replication |
| KEEG | 3.599×10^-3^ | [ko04380](https://www.genome.jp/entry/ko04380) | Cell cycle |
| g: profiler | | | |
| Go Biological Process 2021 | 3.757×10^-10^ | [GO:0006260](https://biit.cs.ut.ee/gprofiler/convert?organism=hsapiens&query=GO:0006260) | DNA replication |
| KEGG | 8.122×10^-8^ | ko03030 | DNA replication |
| Reactome 2022 | 2.594×10^-8^ | R-HSA-69190 | DNA strand elongation |
| Reactome 2022 | 2.413×10^-5^ | R-HSA-1640170 | Cell Cycle |
| Reactome 2022 | 2.415×10^-4^ | R-HSA-69306 | DNA Replication |
| WikiPathway 2023 Human | 3.624×10^-8^ | [WP466](https://biit.cs.ut.ee/gprofiler/convert?organism=hsapiens&query=WP:WP466) | DNA replication |
| WikiPathway 2023 Human | 9.593×10^-3^ | [WP179](https://biit.cs.ut.ee/gprofiler/convert?organism=hsapiens&query=WP:WP179) | Cell cycle |
| [GO: BP](https://maayanlab.cloud/Enrichr/enrich) | 7.059×10^-12^ | [GO:0006259](https://biit.cs.ut.ee/gprofiler/convert?organism=hsapiens&query=GO:0006259) | DNA metabolic process |
|  |  |  |  |
| [GO: BP](https://maayanlab.cloud/Enrichr/enrich) | 4.451×10^-10^ | [GO:0006260](https://biit.cs.ut.ee/gprofiler/convert?organism=hsapiens&query=GO:0006260) | DNA replication |
| GO: CC | 9.829×10^-21^ | [GO:0005654](https://biit.cs.ut.ee/gprofiler/convert?organism=hsapiens&query=GO:0005654) | nucleoplasm |
| GO: CC | 9.322×10^-20^ | [GO:0005737](https://biit.cs.ut.ee/gprofiler/convert?organism=hsapiens&query=GO:0005737) | cytoplasm |
| GO: MF | 1.124×10^-17^ | [GO:0005515](https://biit.cs.ut.ee/gprofiler/convert?organism=hsapiens&query=GO:0005515) | protein binding |
| GO: MF | 2.814×10^-8^ | [GO:0003824](https://biit.cs.ut.ee/gprofiler/convert?organism=hsapiens&query=GO:0003824) | catalytic activity |
