# Supplementary figures and images for: A systems biology approach to find representative genes in Acute Myeloid Leukemia
Source: PLoS One. 2026 Jul 27;21(7):e0352167. doi: 10.1371/journal.pone.0352167 (PMC13405072; doi:10.1371/journal.pone.0352167)

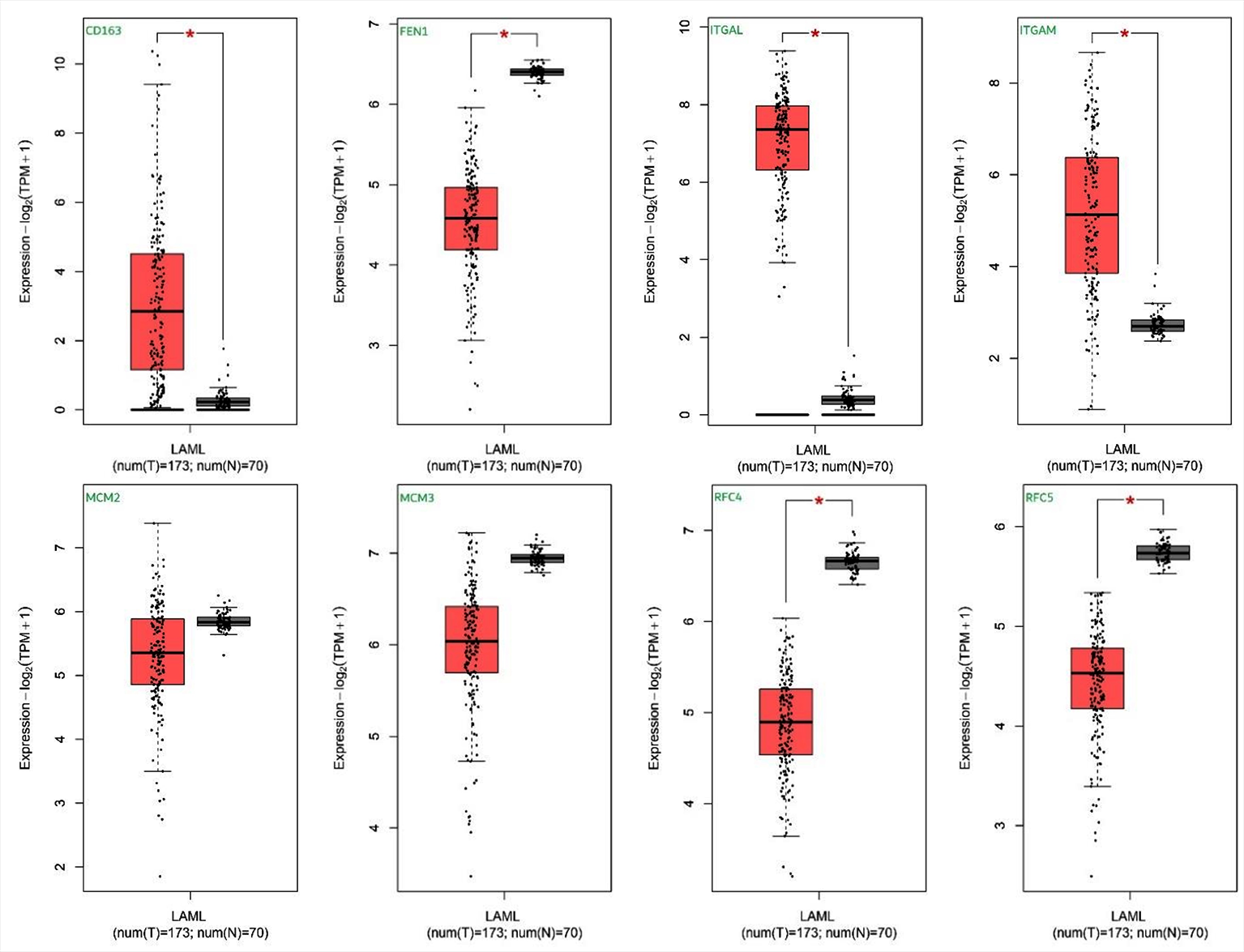

Supplement: S3 Fig — The results are based on TCGA and GTEx data screening in AML (n = 173) and normal control (n = 70) from GEPIA2 database through the Expression DIY (box plot) (*, P < 0.05), the red boxes are represented as the AML sample and the black boxes are represented as the normal tissue. CD163; CD163 molecule, FEN1; flap structure-specific endonuclease 1, ITGAL; integrin subunit alpha L, ITGAM; integrin subunit alpha M, MCM2; minichromosome maintenance complex component 2, MCM3; minichromosome maintenance complex component 3, RFC4; replication factor C subunit 4, RFC5; replication factor C subunit 5, AML, acute myeloid leukemia; TCGA, The Cancer Genome Atlas, GTEx project; Genotype-Tissue Expression. (TIFF) [file pone.0352167.s003.tiff]

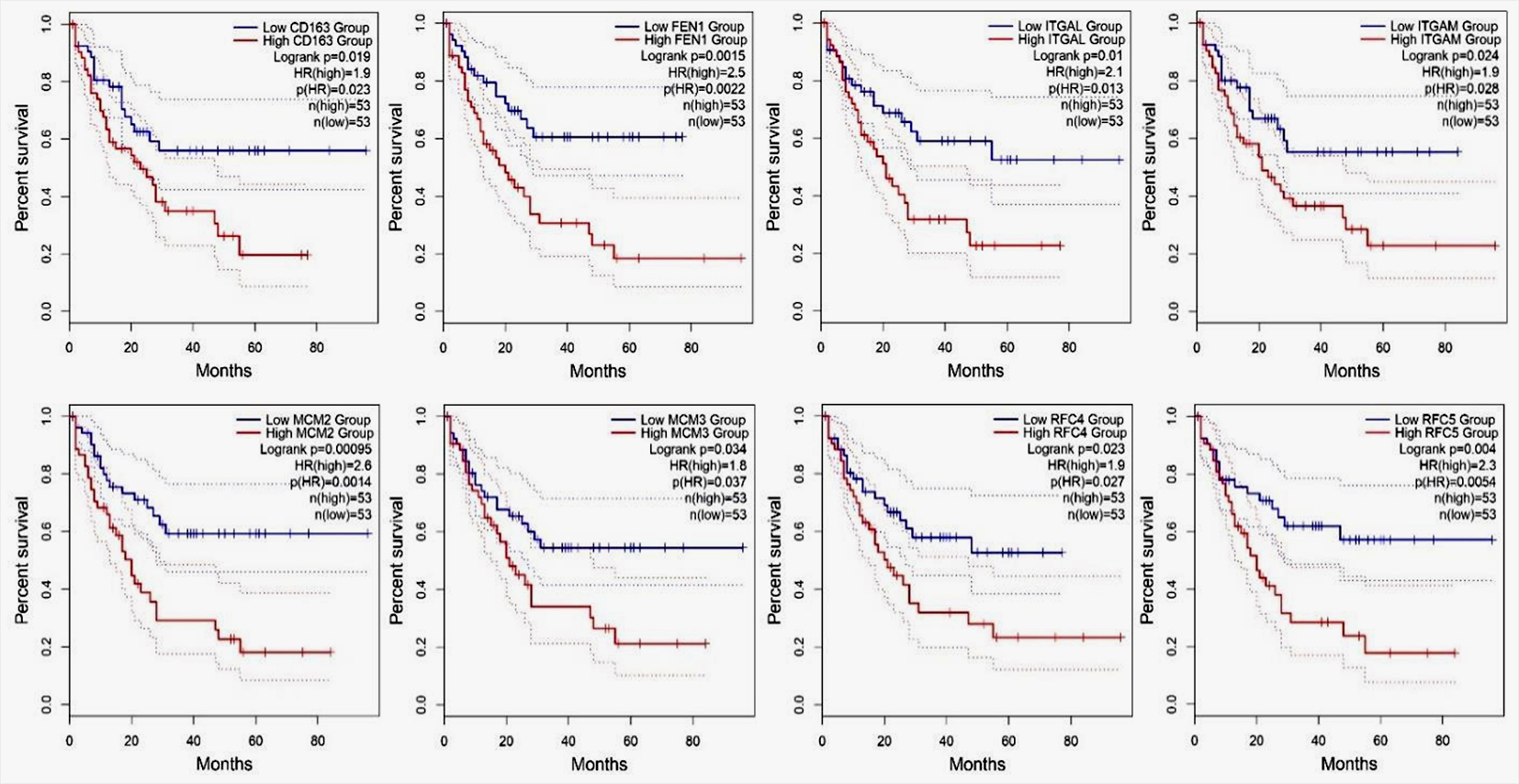

Supplement: S4 Fig — The survival curves are plotted using the GEPIA2 web server. Survival curves are represented as dotted lines, and the solid line represents the 95% confidence interval. The number of AML and normal bone marrow tissues (n) =53. The P values are calculated using log-rank statistics. CD163; CD163 molecule, FEN1; flap structure-specific endonuclease 1, ITGAL; integrin subunit alpha L, ITGAM; integrin subunit alpha M, MCM2; minichromosome maintenance complex component 2, MCM3; minichromosome maintenance complex component 3, RFC4; replication factor C subunit 4, RFC5; replication factor C subunit 5, AML, acute myeloid leukemia; HR, hazard ratio. (TIFF) [file pone.0352167.s004.tiff]
