# Supplementary material for: A systems biology approach to find representative genes in Acute Myeloid Leukemia
Source: PLoS One. 2026 Jul 27;21(7):e0352167. doi: 10.1371/journal.pone.0352167 (PMC13405072; doi:10.1371/journal.pone.0352167)
Supplement: Attachment — Submitted filename: Review comments.docx [file pone.0352167.s005.docx]

This study profiles differentially expressed genes in AML and identifies 8 transcription factors potentially critical for AML development, maintenance, and drug resistance. The intriguing finding that high expression of these genes associates with both drug resistance *and* sensitivity underscores the complexity of drug response in AML. While the computational analysis is valuable, significant revisions are required to strengthen the biological validity and presentation of the findings.

**Major Revisions:**

1. Experimental Validation of Identified TFs: The identification of the 8 key transcription factors relies solely on computational analysis. To confirm their differential expression and biological relevance, the mRNA and protein levels of these TFs must be experimentally validated. This should involve direct comparison between primary AML patient samples and healthy donor samples (e.g., via qRT-PCR and Western blotting/immunohistochemistry).

2. Functional Validation of Drug Resistance Role: The proposed link between these TFs and drug resistance is derived computationally. Functional experiments are essential to support this claim. Perform in vitro and/or in vivo assays (e.g., knockdown/overexpression followed by drug sensitivity testing in AML cell lines or primary cells) to demonstrate a causal role for these TFs in mediating drug resistance or sensitivity.

Minor Revisions:

1. Clarify Data Analysis Workflow: Given the multi-step bioinformatic analysis involving various filters, databases, and tools, include a clear data analysis flow diagram or schematic in the manuscript. This is crucial for transparency and reproducibility.

2. Correct Figure 7 Issues:

Labels Missing:Add the missing labels "a" and "b" to the panels in Figure 7.

Legend Discrepancy:Ensure the legend description for Figure 7a and 7b accurately reflects what is shown in the respective panels (currently described oppositely, lines 233-234).

3. Correct Table 2:Fix the misspelling of "summary statistics" in Table 2.

4. Correct Table 4 Legend:The legend for Table 4 (line 269) states there are 12 genes, but the table lists 24 genes (AML-up and AML-down). Update the legend to accurately reflect the total number of genes presented (24).

5. Clarify Table References:There is confusion regarding Table 5:

*	Line 242 ,249 mentioned Table 5.

*	Confirm and correct all table references.If Table 5 is mentioned in the text, it must exist and be numbered correctly. If it is a typo, correct all instances to refer to the appropriate table (likely Table 4).

6. Revise Figure 9:

Resolution: Provide a high-resolution version of Figure 9, as the current version is illegible.

Legend Accuracy: The current legend description (lines 291-295) does not match the content of Figure 9. Completely revise the legend to accurately describe the data and analysis presented in the figure.

7. Clarify Figure 10: The text (line 309) and Figure 10a list only 7 genes. Confirm whether FEN1 is missing from both the text description and Figure 10a. If FEN1 is part of the analysis, it must be added to both places. If not, ensure the text and figure consistently reference the correct number (7) and list of genes.
