# Supplementary material for: A systems biology approach to find representative genes in Acute Myeloid Leukemia
Source: PLoS One. 2026 Jul 27;21(7):e0352167. doi: 10.1371/journal.pone.0352167 (PMC13405072; doi:10.1371/journal.pone.0352167)
Supplement: Attachment — Submitted filename: Review.docx [file pone.0352167.s006.docx]

The paper presents multi-step bioinformatics study to identify prognostic genes in Acute Myeloid Leukemia (AML) and explore their relationship with drug sensitivity. The authors utilize public gene expression data (GEO-GSE9476) and performs various bioinformatic approaches including differential expression analysis, pathway and network analysis, survival analysis, and finally, drug sensitivity correlation. They identified 8-gene signature (3 upregulated, 5 downregulated) and this is a potentially valuable finding. The use of multiple databases (GEO, GEPIA2, BloodSpot, GDSC) for analysis and validation is also a strength of this paper.

Major comments:

1. Authors have identified MCM2 and MCM3 as genes that are downregulated in AML patients compared to healthy controls. However, when discussing the drug sensitivity results from the CTRP database (Figure 10b), the text states to "MCM2, MCM3 (upregulated in AML patients)". Please clarify.
2. The authors have marked the Ethics Statement as "N/A". This is inappropriate for a study that analyzes human patient and donor data, even if obtained from a public repository. The authors must correct this section to state that the data were obtained from a publicly available source (GEO-GSE9476) and that the original study that generated the data (Stirewalt et al.) received the necessary ethical approvals and patient consent, citing that study.
3. The process for selecting the final list of transcription factors in Table 1 is not detailed. The authors mention using "the statistical significance of the intersections and the repeatability of TFs across various databases". Please provide specific p-value thresholds and a clear definition of "repeatability."
4. The criteria for selecting the hub genes listed in Table 3 are not explicitly stated. It is unclear if they are the top N genes by degree or another centrality metric. This should be clarified.
5. The manuscript states that the 8 identified genes were associated with a "strong prognosis, suggesting high hazard ratios (HR) for AML". A high Hazard Ratio (HR > 1) typically signifies a worse prognosis (i.e., higher risk of a negative event, like death). The term "strong prognosis" is unclear. Please report whether high expression of a gene correlates with better or worse survival.
6. The connection to "epigenetics and lifestyle" is mentioned in the abstract, discussion, and conclusion as a potential factor influencing gene expression and drug response. This is not directly supported by any data in the manuscript. Stating them multiple times without support of data weakens the manuscript.
7. The drug sensitivity data in Figure 10 is confusingly written. For example, "low expression in AML patients leads to reducing resistance” could be written more directly as "low expression in AML patients is associated with increased drug sensitivity." Revising this section to make it more clear is necessary.

The authors have conducted a comprehensive in silico analysis that identifies a promising set of prognostic genes for AML. Please address these issues for paper to be publication ready.
