# Supplementary material for: A systems biology approach to find representative genes in Acute Myeloid Leukemia
Source: PLoS One. 2026 Jul 27;21(7):e0352167. doi: 10.1371/journal.pone.0352167 (PMC13405072; doi:10.1371/journal.pone.0352167)
Supplement: Attachment — Submitted filename: responses to reviewers.docx [file pone.0352167.s008.docx]

**Dear Prof. Purnima Singh  
PLOS ONE**

**Manuscript ID:** PONE-D-25-22273

**Title:** A systems biology approach to find representative genes in Acute Myeloid Leukemia

We sincerely appreciate the time and effort that you and the respected reviewers have dedicated to thoroughly evaluating our manuscript. The insightful comments and constructive feedback have been invaluable in helping us to refine and strengthen our work. We have carefully considered each point raised and have addressed them in detail within manuscript.

Since this study is an *in silico,* systems biology investigation, accordingly, experimental validations fall beyond the scope of the current computational work. therefore, questions regarding the necessity for experimental work (Reviewer #1) were added at the end of manuscript as suggestions for further studies. In the following paragraphs you can find the responses to comments and suggestions of reviewers.

With kindest regards

Khosrow Khalifeh

Reviewer #1:

This study profiles differentially expressed genes in AML and identifies 8 transcription factors potentially critical for AML development, maintenance, and drug resistance. The intriguing finding that high expression of these genes associates with both drug resistance *and* sensitivity underscores the complexity of drug response in AML. While the computational analysis is valuable, significant revisions are required to strengthen the biological validity and presentation of the findings.

Specific comments relating to the text,

**Comment 1:** Experimental Validation of Identified TFs: The identification of the 8 key transcription factors relies solely on computational analysis. To confirm their differential expression and biological relevance, the mRNA and protein levels of these TFs must be experimentally validated. This should involve direct comparison between primary AML patient samples and healthy donor samples (e.g., via qRT-PCR and Western blotting/immunohistochemistry).

**Response:** We sincerely appreciate the respected reviewer for his/her insightful comment which actually led to improvement of our paper.

This study is an *in silico* investigation that uses computational modeling to predict outcomes based on existing experimental data that are available in bioinformatics databases. These predictions are intended to guide the design of future laboratory experiments and to refine or expand previous studies. While experimental validation is indeed critical for confirming the predictions made, such validations fall beyond the scope of the current computational work. However, in line with your valuable feedback, we have incorporated a suggestion at the end of the revised manuscript to encourage further experimental studies to complement and extend the findings of this *in silico* work.

**Comment 2:** Functional Validation of Drug Resistance Role: The proposed link between these TFs and drug resistance is derived computationally. Functional experiments are essential to support this claim. Perform in vitro and/or in vivo assays (e.g., knockdown/overexpression followed by drug sensitivity testing in AML cell lines or primary cells) to demonstrate a causal role for these TFs in mediating drug resistance or sensitivity.

**Response:** we sincerely appreciate your insightful comment regarding the need for experimental support. As mentioned in response to previous comment, this study is an *in silico* investigation, and its primary objective is to generate predictive models based on existing experimental data to guide and optimize future laboratory experiments. Hence, the experimental investigations fall beyond the scope of the current computational work. To address your valuable suggestion, we have emphasized in the revised manuscript the potential experimental approaches that could be pursued to test our predictions, thereby fostering collaboration between computational and experimental research efforts.

**Minor concerns;**

1) Clarify Data Analysis Workflow: Given the multi-step bioinformatic analysis involving various filters, databases, and tools, include a clear data analysis flow diagram or schematic in the manuscript. This is crucial for transparency and reproducibility.

**Response:** This is a good suggestion. We added the overall Workflow of current study at the end of Method section (Figure 1). Accordingly, the numbering of figs was changed.

2. Correct Figure 7 Issues:

Labels Missing: Add the missing labels "a" and "b" to the panels in Figure 7.

Legend Discrepancy:Ensure the legend description for Figure 7a and 7b accurately reflects what is shown in the respective panels (currently described oppositely, lines 233-234).

**Response:** Thank you very much for your careful attention. You are absolutely right. The mentioned points regarding Figure 7 (Figure 8 in the revised version) have been corrected.

3. Correct Table 2: Fix the misspelling of "summary statistics" in Table 2.

**Response:** So many thanks. It has been corrected.

4. Correct Table 4 Legend: The legend for Table 4 (line 269) states there are 12 genes, but the table lists 24 genes (AML-up and AML-down). Update the legend to accurately reflect the total number of genes presented (24).

**Response:** Thank you very much for your insightful attention. You are right. It was corrected.

5. Clarify Table References: There is confusion regarding Table 5:

*	Line 242 ,249 mentioned Table 5.

*	Confirm and correct all table references. If Table 5 is mentioned in the text, it must exist and be numbered correctly. If it is a typo, correct all instances to refer to the appropriate table (likely Table 4).

**Response:** Thank you very much for your important comment. This mistake is due to transferring of some tables into the supplementary files. The Table 5 is indeed Table 3. Thanks to your nice comment this issue has been amended.

6. Revise Figure 9:

Resolution: Provide a high-resolution version of Figure 9, as the current version is illegible.

Legend Accuracy: The current legend description (lines 291-295) does not match the content of Figure 9. Completely revise the legend to accurately describe the data and analysis presented in the figure.

**Response:** Thank you very much for your comment. This image (Fig 10 in the revised version of the manuscript) is the output of the server. Accordingly, we are limited in changing image quality. We have tried to manipulate the image by re-writing the names of the genes and cancer types. We also completely changed the legend of the Figure 10 according to your insightful; comment.

7. Clarify Figure 10: The text (line 309) and Figure 10a list only 7 genes. Confirm whether FEN1 is missing from both the text description and Figure 10a. If FEN1 is part of the analysis, it must be added to both places. If not, ensure the text and figure consistently reference the correct number (7) and list of genes.

**Response:** Thank you very much for your comment, which reflects a thorough and careful reading of the paper. The reason for the absence of the FEN1 gene in the figure is that, based on the database criteria and an FDR of less than 0.05, this gene did not show significant resistance or sensitivity to any of the analyzed drugs. Overall, the concept and interpretation of the figures in this chart are based on the CTRP and GDSC databases, relying on the following two assumptions:

1) Positive correlation indicates a higher gene expression may lead to drug resistance.

2) Negative correlation indicates a higher gene expression may make drug sensitive

Considering your comment, we added an explanatory statement in the Figure legend (Figure 11 in the revised version of the manuscript).

Reviewer #2:

The paper presents multi-step bioinformatics study to identify prognostic genes in Acute Myeloid Leukemia (AML) and explore their relationship with drug sensitivity. The authors utilize public gene expression data (GEO-GSE9476) and performs various bioinformatic approaches including differential expression analysis, pathway and network analysis, survival analysis, and finally, drug sensitivity correlation. They identified 8-gene signature (3 upregulated, 5 downregulated) and this is a potentially valuable finding. The use of multiple databases (GEO, GEPIA2, BloodSpot, GDSC) for analysis and validation is also a strength of this paper.

**Comment 1:** Authors have identified MCM2 and MCM3 as genes that are downregulated in AML patients compared to healthy controls. However, when discussing the drug sensitivity results from the CTRP database (Figure 10b), the text states to "MCM2, MCM3 (upregulated in AML patients)". Please clarify.

**Response:** Thank you very much for your careful attention. You are absolutely right. It was corrected.

**Comment 2:** The authors have marked the Ethics Statement as "N/A". This is inappropriate for a study that analyzes human patient and donor data, even if obtained from a public repository. The authors must correct this section to state that the data were obtained from a publicly available source (GEO-GSE9476) and that the original study that generated the data (Stirewalt et al.) received the necessary ethical approvals and patient consent, citing that study.

**Response:** Thank you very much for your nice comment. Thanks to your experience, it will be amended during submission process.

**Comment 3:** The process for selecting the final list of transcription factors in Table 1 is not detailed. The authors mention using "the statistical significance of the intersections and the repeatability of TFs across various databases". Please provide specific p-value thresholds and a clear definition of "repeatability."

**Response:** Thank you very much for your important comment. The missing information was added into the material and method section.

**Comment 4:** The criteria for selecting the hub genes listed in Table 3 are not explicitly stated. It is unclear if they are the top N genes by degree or another centrality metric. This should be clarified.

**Response:** So many thanks for your insightful comment. In line with your comment, we added more explanatory statements in the revised version of the manuscript (Section 3.4.1. Hub Genes and Significant Modules of the PPI Network)

**Comment 5:** The manuscript states that the 8 identified genes were associated with a "strong prognosis, suggesting high hazard ratios (HR) for AML". A high Hazard Ratio (HR > 1) typically signifies a worse prognosis (i.e., higher risk of a negative event, like death). The term "strong prognosis" is unclear. Please report whether high expression of a gene correlates with better or worse survival.

**Response:** Thank you very much for your suggestion. You are right. The current statement is confusing for readers. We applied some changes in the corresponding paragraph. The Legend of the Figure 10 has also changed according to your comment.

**Comment 6:** The connection to "epigenetics and lifestyle" is mentioned in the abstract, discussion, and conclusion as a potential factor influencing gene expression and drug response. This is not directly supported by any data in the manuscript. Stating them multiple times without support of data weakens the manuscript.

**Response:** Thank you very much for your insightful comment. Indeed, our aim was to extend the results of recent modeling studies—specifically those concerning the influence of expression patterns of certain genes on AML—toward a more generalized biological reality to provide a biologically meaningful interpretation of these findings. To this end, we discussed how gene expression patterns may be influenced by environmental factors. However, as you correctly pointed out, such a conclusion cannot be drawn directly or with absolute certainty. Therefore, in response to your comment, we have softened the wording of this conclusion and avoided any deterministic interpretation. Also, the final respective statement at the conclusion section was deleted.

**Comment 7:** The drug sensitivity data in Figure 10 is confusingly written. For example, "low expression in AML patients leads to reducing resistance” could be written more directly as "low expression in AML patients is associated with increased drug sensitivity." Revising this section to make it more clear is necessary.

**Response:** Thank you for your suggestion. You are absolutely right. The paragraph was rewritten.

The authors have conducted a comprehensive in silico analysis that identifies a promising set of prognostic genes for AML. Please address these issues for paper to be publication ready.

We believe that the paper significancy improved by considering your insightful comments and suggestions. Thank you again for saving your time and details to read the manuscript.
