# Supplementary material for: A systems biology approach to find representative genes in Acute Myeloid Leukemia
Source: PLoS One. 2026 Jul 27;21(7):e0352167. doi: 10.1371/journal.pone.0352167 (PMC13405072; doi:10.1371/journal.pone.0352167)
Supplement: Attachment — Submitted filename: comments.docx [file pone.0352167.s009.docx]

The number of patient samples is relatively low for analysis (Page 4). Although this limitation is acknowledged, the study still provides valuable insights.
Since this is a computational study, experimental validation is not required within its current scope; however, suggesting future *in vivo* and *in vitro* experiments would further strengthen the work. In the *Discussion* section, the importance of future *in vitro* and *in vivo* validation (e.g., qRT-PCR, Western blotting, or functional assays) to confirm the roles of these transcription factors (TFs) in AML progression and drug response has been appropriately explained.

The manuscript can be accepted after verifying the following minor corrections:

- In the *Methods* section, ensure a clear distinction between the microarray platform (GEO-GPL96) and the dataset (GEO-GSE9476).
- In the *Methods* section, include explicit details regarding the statistical thresholds (p-values) and the criteria for “repeatability” used in transcription factor selection.
- Verify the consistency in the expression status of **MCM2** and **MCM3** between the main text and the drug sensitivity figure, and correct if necessary for accuracy.
- Clarify the **hub gene selection process** in Section 3.4.1 by specifying the **centrality metrics** used.
- Confirm that the **interpretation of survival results** has been revised to clearly indicate whether higher gene expression correlates with better or worse prognosis.
