# Supplementary material for: A systems biology approach to find representative genes in Acute Myeloid Leukemia
Source: PLoS One. 2026 Jul 27;21(7):e0352167. doi: 10.1371/journal.pone.0352167 (PMC13405072; doi:10.1371/journal.pone.0352167)
Supplement: Attachment — Submitted filename: responses_to_reviewers_auresp_3.docx [file pone.0352167.s011.docx]

**Dear Editor  
PLOS ONE**

**Manuscript ID:** PONE-D-25-22273R2

**Title:** A systems biology approach to find representative genes in Acute Myeloid Leukemia

We sincerely appreciate the time and effort that you and the respected reviewer have dedicated to thoroughly evaluating our manuscript. We have carefully considered each point raised, and have addressed them in detail within manuscript. All changes in the final version of the manuscript were highlighted.

With kindest regards

Khosrow Khalifeh

comment 1:

The manuscript can be accepted after verifying the following minor corrections:

1) In the Methods section, ensure a clear distinction between the microarray platform (GEO-GPL96) and the dataset (GEO-GSE9476).

**Response:**

We thank the respected Editor for highlighting the need for clarity regarding the use of GEO-GPL96 and GEO-GSE9476 in our study. More explanation was added in the main text of the manuscript. (Pages 4 and 5)

comment 2: In the Methods section, include explicit details regarding the statistical thresholds (p-values) and the criteria for “repeatability” used in transcription factor selection.

**Response:** Thank you very much for your suggestion. The details of statistical criteria were added into the manuscript. (Page 7)

comment 3: Verify the consistency in the expression status of MCM2 and MCM3 between the main text and the drug sensitivity figure, and correct if necessary for accuracy.

**Response:** So many thanks for your comment. We added more statements to clarify our explanations. (Page 17)

comment 4: Clarify the hub gene selection process in Section 3.4.1 by specifying the centrality metrics used.

**Response:** Thank you very much for your suggestion. More explanatory statements were added into the revised version of manuscript. (Page 12)

comment 5: Confirm that the interpretation of survival results has been revised to clearly indicate whether higher gene expression correlates with better or worse prognosis.

**Response:** Thank you very much for your comment. We clarified our conclusion by adding more statements into the manuscript. (Pages 17 and 18)
