# Supplementary material for: A systems biology approach to find representative genes in Acute Myeloid Leukemia
Source: PLoS One. 2026 Jul 27;21(7):e0352167. doi: 10.1371/journal.pone.0352167 (PMC13405072; doi:10.1371/journal.pone.0352167)
Supplement: Peer review history

# Peer review: Decision Letter 0

**DOI:** 10.1371/journal.pone.0352167.r001

**Authors:** Purnima Singh

29 Jul 2025

PONE-D-25-22273

A systems biology approach to find representative genes in Acute Myeloid Leukemia

PLOS ONE

Dear Dr. Kalifeh,

Thank you for submitting your manuscript to PLOS ONE. After careful consideration, we feel that it has merit but does not fully meet PLOS ONE’s publication criteria as it currently stands. Therefore, we invite you to submit a revised version of the manuscript that addresses the points raised during the review process.

Please submit your revised manuscript by Sep 12 2025 11:59PM. If you will need more time than this to complete your revisions, please reply to this message or contact the journal office at plosone@plos.org. When you're ready to submit your revision, log on to https://www.editorialmanager.com/pone/ and select the 'Submissions Needing Revision' folder to locate your manuscript file.

Please include the following items when submitting your revised manuscript:

- A rebuttal letter that responds to each point raised by the academic editor and reviewer(s). You should upload this letter as a separate file labeled 'Response to Reviewers'.
- A marked-up copy of your manuscript that highlights changes made to the original version. You should upload this as a separate file labeled 'Revised Manuscript with Track Changes'.
- An unmarked version of your revised paper without tracked changes. You should upload this as a separate file labeled 'Manuscript'.

If you would like to make changes to your financial disclosure, please include your updated statement in your cover letter. Guidelines for resubmitting your figure files are available below the reviewer comments at the end of this letter.

If applicable, we recommend that you deposit your laboratory protocols in protocols.io to enhance the reproducibility of your results. Protocols.io assigns your protocol its own identifier (DOI) so that it can be cited independently in the future. For instructions see: https://journals.plos.org/plosone/s/submission-guidelines#loc-laboratory-protocols. Additionally, PLOS ONE offers an option for publishing peer-reviewed Lab Protocol articles, which describe protocols hosted on protocols.io. Read more information on sharing protocols at https://plos.org/protocols?utm_medium=editorial-email&utm_source=authorletters&utm_campaign=protocols.

We look forward to receiving your revised manuscript.

Kind regards,

Purnima Singh, PhD

Academic Editor

PLOS ONE

**Journal Requirements:**

1. When submitting your revision, we need you to address these additional requirements.

Please ensure that your manuscript meets PLOS ONE's style requirements, including those for file naming. The PLOS ONE style templates can be found at

https://journals.plos.org/plosone/s/file?id=wjVg/PLOSOne_formatting_sample_main_body.pdf and

https://journals.plos.org/plosone/s/file?id=ba62/PLOSOne_formatting_sample_title_authors_affiliations.pdf

2. Please note that PLOS ONE has specific guidelines on code sharing for submissions in which author-generated code underpins the findings in the manuscript. In these cases, we expect all author-generated code to be made available without restrictions upon publication of the work. Please review our guidelines at https://journals.plos.org/plosone/s/materials-and-software-sharing#loc-sharing-code and ensure that your code is shared in a way that follows best practice and facilitates reproducibility and reuse.

3. When completing the data availability statement of the submission form, you indicated that you will make your data available on acceptance. We strongly recommend all authors decide on a data sharing plan before acceptance, as the process can be lengthy and hold up publication timelines. Please note that, though access restrictions are acceptable now, your entire data will need to be made freely accessible if your manuscript is accepted for publication. This policy applies to all data except where public deposition would breach compliance with the protocol approved by your research ethics board. If you are unable to adhere to our open data policy, please kindly revise your statement to explain your reasoning and we will seek the editor's input on an exemption. Please be assured that, once you have provided your new statement, the assessment of your exemption will not hold up the peer review process.

4. Please upload a new copy of Figures 5 and 6, as the detail is not clear. Please follow the link for more information: https://blogs.plos.org/plos/2019/06/looking-good-tips-for-creating-your-plos-figures-graphics/" https://blogs.plos.org/plos/2019/06/looking-good-tips-for-creating-your-plos-figures-graphics/

5. If the reviewer comments include a recommendation to cite specific previously published works, please review and evaluate these publications to determine whether they are relevant and should be cited. There is no requirement to cite these works unless the editor has indicated otherwise.

**Additional Editor Comments:**

The authors mention that "two microarray datasets, GEO-GPL96 (Nov, 2007) and its last update GEO-GSE9476 (Nov 2018), were found to be compatible with our filtering parameters." However, according to the GEO database, GEO-GPL96 provides a description of the array technology, specifically detailing the platform's design by Affymetrix. This description suggests that the dataset might be primarily related to array design and not specifically focused on the data used in the gene expression analysis, which is important to clarify.

On the other hand, GSE9476 corresponds to a dataset involving gene expression profiles from normal hematopoietic cells (38 healthy donors) and leukemic blasts (26 AML patients). The data includes various cell types, such as CD34+ selected cells (N = 18), unselected bone marrow cells (N = 10), and unselected peripheral blood cells (N = 10). Therefore, it would be important to distinguish between the description of the platform (GEO-GPL96) and the specific dataset (GSE9476).

The authors must italicize human gene names throughout the manuscript to ensure consistency with nomenclature guidelines.

[Note: HTML markup is below. Please do not edit.]

Reviewers' comments:

Reviewer's Responses to Questions

**Comments to the Author**

1. Is the manuscript technically sound, and do the data support the conclusions?

The manuscript must describe a technically sound piece of scientific research with data that supports the conclusions. Experiments must have been conducted rigorously, with appropriate controls, replication, and sample sizes. The conclusions must be drawn appropriately based on the data presented.

Reviewer #1: Partly

Reviewer #2: Yes

**********

2. Has the statistical analysis been performed appropriately and rigorously?

Reviewer #1: N/A

Reviewer #2: Yes

**********

3. Have the authors made all data underlying the findings in their manuscript fully available?

The PLOS Data policy requires authors to make all data underlying the findings described in their manuscript fully available without restriction, with rare exception (please refer to the Data Availability Statement in the manuscript PDF file). The data should be provided as part of the manuscript or its supporting information, or deposited to a public repository. For example, in addition to summary statistics, the data points behind means, medians and variance measures should be available. If there are restrictions on publicly sharing data—e.g. participant privacy or use of data from a third party—those must be specified.

Reviewer #1: No

Reviewer #2: Yes

**********

4. Is the manuscript presented in an intelligible fashion and written in standard English?

PLOS ONE does not copyedit accepted manuscripts, so the language in submitted articles must be clear, correct, and unambiguous. Any typographical or grammatical errors should be corrected at revision, so please note any specific errors here.

Reviewer #1: Yes

Reviewer #2: Yes

**********

5. Review Comments to the Author

Please use the space provided to explain your answers to the questions above. You may also include additional comments for the author, including concerns about dual publication, research ethics, or publication ethics. (Please upload your review as an attachment if it exceeds 20,000 characters)

**Reviewer #1:**  This study profiles differentially expressed genes in AML and identifies 8 transcription factors potentially critical for AML development, maintenance, and drug resistance. The intriguing finding that high expression of these genes associates with both drug resistance *and* sensitivity underscores the complexity of drug response in AML. While the computational analysis is valuable, significant revisions are required to strengthen the biological validity and presentation of the findings.

Major Revisions:

1. Experimental Validation of Identified TFs: The identification of the 8 key transcription factors relies solely on computational analysis. To confirm their differential expression and biological relevance, the mRNA and protein levels of these TFs must be experimentally validated. This should involve direct comparison between primary AML patient samples and healthy donor samples (e.g., via qRT-PCR and Western blotting/immunohistochemistry).

2. Functional Validation of Drug Resistance Role: The proposed link between these TFs and drug resistance is derived computationally. Functional experiments are essential to support this claim. Perform in vitro and/or in vivo assays (e.g., knockdown/overexpression followed by drug sensitivity testing in AML cell lines or primary cells) to demonstrate a causal role for these TFs in mediating drug resistance or sensitivity.

Minor Revisions:

1. Clarify Data Analysis Workflow: Given the multi-step bioinformatic analysis involving various filters, databases, and tools, include a clear data analysis flow diagram or schematic in the manuscript. This is crucial for transparency and reproducibility.

2. Correct Figure 7 Issues:

Labels Missing:Add the missing labels "a" and "b" to the panels in Figure 7.

Legend Discrepancy:Ensure the legend description for Figure 7a and 7b accurately reflects what is shown in the respective panels (currently described oppositely, lines 233-234).

3. Correct Table 2:Fix the misspelling of "summary statistics" in Table 2.

4. Correct Table 4 Legend:The legend for Table 4 (line 269) states there are 12 genes, but the table lists 24 genes (AML-up and AML-down). Update the legend to accurately reflect the total number of genes presented (24).

5. Clarify Table References:There is confusion regarding Table 5:

* Line 242 ,249 mentioned Table 5.

* Confirm and correct all table references.If Table 5 is mentioned in the text, it must exist and be numbered correctly. If it is a typo, correct all instances to refer to the appropriate table (likely Table 4).

6. Revise Figure 9:

Resolution: Provide a high-resolution version of Figure 9, as the current version is illegible.

Legend Accuracy: The current legend description (lines 291-295) does not match the content of Figure 9. Completely revise the legend to accurately describe the data and analysis presented in the figure.

7. Clarify Figure 10: The text (line 309) and Figure 10a list only 7 genes. Confirm whether FEN1 is missing from both the text description and Figure 10a. If FEN1 is part of the analysis, it must be added to both places. If not, ensure the text and figure consistently reference the correct number (7) and list of genes.

**Reviewer #2:**  Please revise the manuscript based on comments. Some of the statistical figures are missing in the paper and inclusion of them would be appreciated. Some clarifications on threshold is also needed to support the conclusions.

**********

6. PLOS authors have the option to publish the peer review history of their article (what does this mean?). If published, this will include your full peer review and any attached files.

If you choose “no”, your identity will remain anonymous but your review may still be made public.

**Do you want your identity to be public for this peer review?**  For information about this choice, including consent withdrawal, please see our Privacy Policy.

Reviewer #1: No

Reviewer #2: No

**********

[NOTE: If reviewer comments were submitted as an attachment file, they will be attached to this email and accessible via the submission site. Please log into your account, locate the manuscript record, and check for the action link "View Attachments". If this link does not appear, there are no attachment files.]

While revising your submission, please upload your figure files to the Preflight Analysis and Conversion Engine (PACE) digital diagnostic tool, https://pacev2.apexcovantage.com/. PACE helps ensure that figures meet PLOS requirements. To use PACE, you must first register as a user. Registration is free. Then, login and navigate to the UPLOAD tab, where you will find detailed instructions on how to use the tool. If you encounter any issues or have any questions when using PACE, please email PLOS at figures@plos.org. Please note that Supporting Information files do not need this step.

Attachment Submitted filename: Review comments.docx

Attachment Submitted filename: Review.docx

# Author comment: Author response to Decision Letter 1

**DOI:** 10.1371/journal.pone.0352167.r002

26 Aug 2025

Dear Prof. Purnima Singh

PLOS ONE

Manuscript ID: PONE-D-25-22273

Title: A systems biology approach to find representative genes in Acute Myeloid Leukemia

We sincerely appreciate the time and effort that you and the respected reviewers have dedicated to thoroughly evaluating our manuscript. The insightful comments and constructive feedback have been invaluable in helping us to refine and strengthen our work. We have carefully considered each point raised and have addressed them in detail within manuscript.

Since this study is an in silico, systems biology investigation, accordingly, experimental validations fall beyond the scope of the current computational work. therefore, questions regarding the necessity for experimental work (Reviewer #1) were added at the end of manuscript as suggestions for further studies. In the following paragraphs you can find the responses to comments and suggestions of reviewers.

With kindest regards

Khosrow Khalifeh

________________________________________

Reviewer #1:

This study profiles differentially expressed genes in AML and identifies 8 transcription factors potentially critical for AML development, maintenance, and drug resistance. The intriguing finding that high expression of these genes associates with both drug resistance *and* sensitivity underscores the complexity of drug response in AML. While the computational analysis is valuable, significant revisions are required to strengthen the biological validity and presentation of the findings.

Specific comments relating to the text,

Comment 1: Experimental Validation of Identified TFs: The identification of the 8 key transcription factors relies solely on computational analysis. To confirm their differential expression and biological relevance, the mRNA and protein levels of these TFs must be experimentally validated. This should involve direct comparison between primary AML patient samples and healthy donor samples (e.g., via qRT-PCR and Western blotting/immunohistochemistry).

Response: We sincerely appreciate the respected reviewer for his/her insightful comment which actually led to improvement of our paper.

This study is an in silico investigation that uses computational modeling to predict outcomes based on existing experimental data that are available in bioinformatics databases. These predictions are intended to guide the design of future laboratory experiments and to refine or expand previous studies. While experimental validation is indeed critical for confirming the predictions made, such validations fall beyond the scope of the current computational work. However, in line with your valuable feedback, we have incorporated a suggestion at the end of the revised manuscript to encourage further experimental studies to complement and extend the findings of this in silico work.

Comment 2: Functional Validation of Drug Resistance Role: The proposed link between these TFs and drug resistance is derived computationally. Functional experiments are essential to support this claim. Perform in vitro and/or in vivo assays (e.g., knockdown/overexpression followed by drug sensitivity testing in AML cell lines or primary cells) to demonstrate a causal role for these TFs in mediating drug resistance or sensitivity.

Response: we sincerely appreciate your insightful comment regarding the need for experimental support. As mentioned in response to previous comment, this study is an in silico investigation, and its primary objective is to generate predictive models based on existing experimental data to guide and optimize future laboratory experiments. Hence, the experimental investigations fall beyond the scope of the current computational work. To address your valuable suggestion, we have emphasized in the revised manuscript the potential experimental approaches that could be pursued to test our predictions, thereby fostering collaboration between computational and experimental research efforts.

Minor concerns;

1) Clarify Data Analysis Workflow: Given the multi-step bioinformatic analysis involving various filters, databases, and tools, include a clear data analysis flow diagram or schematic in the manuscript. This is crucial for transparency and reproducibility.

Response: This is a good suggestion. We added the overall Workflow of current study at the end of Method section (Figure 1). Accordingly, the numbering of figs was changed.

2. Correct Figure 7 Issues:

Labels Missing: Add the missing labels "a" and "b" to the panels in Figure 7.

Legend Discrepancy:Ensure the legend description for Figure 7a and 7b accurately reflects what is shown in the respective panels (currently described oppositely, lines 233-234).

Response: Thank you very much for your careful attention. You are absolutely right. The mentioned points regarding Figure 7 (Figure 8 in the revised version) have been corrected.

3. Correct Table 2: Fix the misspelling of "summary statistics" in Table 2.

Response: So many thanks. It has been corrected.

4. Correct Table 4 Legend: The legend for Table 4 (line 269) states there are 12 genes, but the table lists 24 genes (AML-up and AML-down). Update the legend to accurately reflect the total number of genes presented (24).

Response: Thank you very much for your insightful attention. You are right. It was corrected.

5. Clarify Table References: There is confusion regarding Table 5:

* Line 242 ,249 mentioned Table 5.

* Confirm and correct all table references. If Table 5 is mentioned in the text, it must exist and be numbered correctly. If it is a typo, correct all instances to refer to the appropriate table (likely Table 4).

Response: Thank you very much for your important comment. This mistake is due to transferring of some tables into the supplementary files. The Table 5 is indeed Table 3. Thanks to your nice comment this issue has been amended.

6. Revise Figure 9:

Resolution: Provide a high-resolution version of Figure 9, as the current version is illegible.

Legend Accuracy: The current legend description (lines 291-295) does not match the content of Figure 9. Completely revise the legend to accurately describe the data and analysis presented in the figure.

Response: Thank you very much for your comment. This image (Fig 10 in the revised version of the manuscript) is the output of the server. Accordingly, we are limited in changing image quality. We have tried to manipulate the image by re-writing the names of the genes and cancer types. We also completely changed the legend of the Figure 10 according to your insightful; comment.

7. Clarify Figure 10: The text (line 309) and Figure 10a list only 7 genes. Confirm whether FEN1 is missing from both the text description and Figure 10a. If FEN1 is part of the analysis, it must be added to both places. If not, ensure the text and figure consistently reference the correct number (7) and list of genes.

Response: Thank you very much for your comment, which reflects a thorough and careful reading of the paper. The reason for the absence of the FEN1 gene in the figure is that, based on the database criteria and an FDR of less than 0.05, this gene did not show significant resistance or sensitivity to any of the analyzed drugs. Overall, the concept and interpretation of the figures in this chart are based on the CTRP and GDSC databases, relying on the following two assumptions:

1) Positive correlation indicates a higher gene expression may lead to drug resistance.

2) Negative correlation indicates a higher gene expression may make drug sensitive

Considering your comment, we added an explanatory statement in the Figure legend (Figure 11 in the revised version of the manuscript).

Reviewer #2:

The paper presents multi-step bioinformatics study to identify prognostic genes in Acute Myeloid Leukemia (AML) and explore their relationship with drug sensitivity. The authors utilize public gene expression data (GEO-GSE9476) and performs various bioinformatic approaches including differential expression analysis, pathway and network analysis, survival analysis, and finally, drug sensitivity correlation. They identified 8-gene signature (3 upregulated, 5 downregulated) and this is a potentially valuable finding. The use of multiple databases (GEO, GEPIA2, BloodSpot, GDSC) for analysis and validation is also a strength of this paper.

Comment 1: Authors have identified MCM2 and MCM3 as genes that are downregulated in AML patients compared to healthy controls. However, when discussing the drug sensitivity results from the CTRP database (Figure 10b), the text states to "MCM2, MCM3 (upregulated in AML patients)". Please clarify.

Response: Thank you very much for your careful attention. You are absolutely right. It was corrected.

Comment 2: The authors have marked the Ethics Statement as "N/A". This is inappropriate for a study that analyzes human patient and donor data, even if obtained from a public repository. The authors must correct this section to state that the data were obtained from a publicly available source (GEO-GSE9476) and that the original study that generated the data (Stirewalt et al.) received the necessary ethical approvals and patient consent, citing that study.

Response: Thank you very much for your nice comment. Thanks to your experience, it will be amended during submission process.

Comment 3: The process for selecting the final list of transcription factors in Table 1 is not detailed. The authors mention using "the statistical significance of the intersections and the repeatability of TFs across various databases". Please provide specific p-value thresholds and a clear definition of "repeatability."

Response: Thank you very much for your important comment. The missing information was added into the material and method section.

Comment 4: The criteria for selecting the hub genes listed in Table 3 are not explicitly stated. It is unclear if they are the top N genes by degree or another centrality metric. This should be clarified.

Response: So many thanks for your insightful comment. In line with your comment, we added more explanatory statements in the revised version of the manuscript (Section 3.4.1. Hub Genes and Significant Modules of the PPI Network)

Comment 5: The manuscript states that the 8 identified genes were associated with a "strong prognosis, suggesting high hazard ratios (HR) for AML". A high Hazard Ratio (HR > 1) typically signifies a worse prognosis (i.e., higher risk of a negative event, like death). The term "strong prognosis" is unclear. Please report whether high expression of a gene correlates with better or worse survival.

Response: Thank you very much for your suggestion. You are right. The current statement is confusing for readers. We applied some changes in the corresponding paragraph. The Legend of the Figure 10 has also changed according to your comment.

Comment 6: The connection to "epigenetics and lifestyle" is mentioned in the abstract, discussion, and conclusion as a potential factor influencing gene expression and drug response. This is not directly supported by any data in the manuscript. Stating them multiple times without support of data weakens the manuscript.

Response: Thank you very much for your insightful comment. Indeed, our aim was to extend the results of recent modeling studies—specifically those concerning the influence of expression patterns of certain genes on AML—toward a more generalized biological reality to provide a biologically meaningful interpretation of these findings. To this end, we discussed how gene expression patterns may be influenced by environmental factors. However, as you correctly pointed out, such a conclusion cannot be drawn directly or with absolute certainty. Therefore, in response to your comment, we have softened the wording of this conclusion and avoided any deterministic interpretation. Also, the final respective statement at the conclusion section was deleted.

Comment 7: The drug sensitivity data in Figure 10 is confusingly written. For example, "low expression in AML patients leads to reducing resistance” could be written more directly as "low expression in AML patients is associated with increased drug sensitivity." Revising this section to make it more clear is necessary.

Response: Thank you for your suggestion. You are absolutely right. The paragraph was rewritten.

The authors have conducted a comprehensive in silico analysis that identifies a promising set of prognostic genes for AML. Please address these issues for paper to be publication ready.

We believe that the paper significancy improved by considering your insightful comments and suggestions. Thank you again for saving your time and details to read the manuscript.

Attachment Submitted filename: responses to reviewers.docx

# Editor report: Decision Letter 1

**DOI:** 10.1371/journal.pone.0352167.r003

**Authors:** Purnima Singh, Mohammad H. Ghazimoradi

4 Sep 2025

PONE-D-25-22273R1

A systems biology approach to find representative genes in Acute Myeloid Leukemia

PLOS ONE

Dear Dr. Kalifeh,

Thank you for submitting your manuscript to PLOS ONE. After careful consideration, we have decided that your manuscript does not meet our criteria for publication and must therefore be rejected.

I am sorry that we cannot be more positive on this occasion, but hope that you appreciate the reasons for this decision.

Kind regards,

Mohammad H. Ghazimoradi

Academic Editor

PLOS ONE

Additional Editor Comments :

While the work is interesting, without proper experimental work as the reviewer ask, the manuscript is not suitable for publication.

[Note: HTML markup is below. Please do not edit.]

[NOTE: If reviewer comments were submitted as an attachment file, they will be attached to this email and accessible via the submission site. Please log into your account, locate the manuscript record, and check for the action link "View Attachments". If this link does not appear, there are no attachment files.]

- - - - -

For journal use only: PONEDEC3

# Author comment: Author response to Decision Letter 2

**DOI:** 10.1371/journal.pone.0352167.r004

12 Sep 2025

Subject: Appeal for Reconsideration of Manuscript

Title: A systems biology approach to find representative genes in Acute Myeloid Leukemia

Manuscript ID: PONE-D-25-22273R2

Dear Purnima Singh, Academic Editor, PLOS ONE

At first, I would like to thank you for saving your times and details to manage our paper. We Also thanks the respected reviewers for the valuable comments and suggestions that help us to improve the paper.

As you know, our manuscript was initially submitted to PLOS ONE and underwent peer review, where both reviewers provided positive feedback and recommended revisions. We addressed all comments from the reviewers in a thorough revision, submitting the revised version along with a detailed point-by-point response. However, after submission, we received notification that the original handling editor was unavailable, and the manuscript was reassigned to a new editor who is expert in the experimental Biology rather than computational one. Regrettably, the new editor rejected the manuscript within a few hours, citing the absence of an experimental laboratory section as the primary reason. We believe this decision was made without consulting the reviewers or considering the fact that this work is classified in the area of Modelling and Computational Biology. This work aligns with PLOS ONE's scope, which emphasizes innovative research across disciplines, including computational biology, without mandating experimental validation in all cases. The rejection rationale does not adequately account for the nature of our study or the positive assessments from the initial peer review. We kindly request that you reconsider this decision by assigning the manuscript to an expert editor in the area of computational Biology and allowing it to proceed to re-review by the original referees, who are best positioned to evaluate the revisions. We are confident that the revised manuscript meets PLOS ONE's criteria for publication and contributes meaningfully to the field. Thank you for considering this appeal. We value the opportunity to contribute to PLOS ONE and look forward to your response. You can find below the point-by-point responses to the reviewer’s comments.

Responses to Editor and Reviewer Comments

Editor comment:

We appreciate the opportunity to improve the clarity of our manuscript.

Editor comment 1) The authors mention that "two microarray datasets, GEO-GPL96 (Nov, 2007) and its last update GEO-GSE9476 (Nov 2018), were found to be compatible with our filtering parameters." However, according to the GEO database, GEO-GPL96 provides a description of the array technology, specifically detailing the platform's design by Affymetrix. This description suggests that the dataset might be primarily related to array design and not specifically focused on the data used in the gene expression analysis, which is important to clarify.

On the other hand, GSE9476 corresponds to a dataset involving gene expression profiles from normal hematopoietic cells (38 healthy donors) and leukemic blasts (26 AML patients). The data includes various cell types, such as CD34+ selected cells (N = 18), unselected bone marrow cells (N = 10), and unselected peripheral blood cells (N = 10). Therefore, it would be important to distinguish between the description of the platform (GEO-GPL96) and the specific dataset (GSE9476).

Response:

We thank the respected Editor for highlighting the need for clarity regarding the use of GEO-GPL96 and GEO-GSE9476 in our study. To address this, we acknowledge that GEO-GPL96 refers to the Affymetrix platform description, which outlines the microarray technology and probe design used for gene expression profiling. In contrast, GEO-GSE9476 is the specific dataset containing gene expression profiles from normal hematopoietic cells (38 healthy donors) and leukemic blasts (26 AML patients), including CD34+ selected cells (N=18), unselected bone marrow cells (N=10), and unselected peripheral blood cells (N=10).

In our manuscript, we used GEO-GPL96 to confirm the compatibility of the array platform with our filtering parameters, ensuring that the probe design and technical specifications aligned with our analysis pipeline. The actual gene expression data analyzed in our study were derived from GEO-GSE9476, which provided the relevant biological samples for our AML modeling. To avoid confusion, we have revised the manuscript to clearly distinguish between GEO-GPL96 as the platform description and GEO-GSE9476 as the dataset used for gene expression analysis. Specifically, we have updated the text in the Methods section to read:

Gene expression data were obtained from GEO-GSE9476 (last updated Nov 2018), which includes profiles from normal hematopoietic cells (N=38) and leukemic blasts (N=26) across various cell types (CD34+ selected cells, N=18; unselected bone marrow cells, N=10; unselected peripheral blood cells, N=10). The dataset was generated using the Affymetrix platform described in GEO-GPL96 (Nov 2007), which was verified to meet our filtering parameters for probe design and data compatibility.

We believe this revision clarifies the distinct roles of GEO-GPL96 and GEO-GSE9476 in our study and addresses your concern.

Editor comment 2): The authors must italicize human gene names throughout the manuscript to ensure consistency with nomenclature guidelines.

Response: Thank you very much for your suggestion. The gene names italicized throughout the manuscript.

Reviewer #1:

This study profiles differentially expressed genes in AML and identifies 8 transcription factors potentially critical for AML development, maintenance, and drug resistance. The intriguing finding that high expression of these genes associates with both drug resistance *and* sensitivity underscores the complexity of drug response in AML. While the computational analysis is valuable, significant revisions are required to strengthen the biological validity and presentation of the findings.

Specific comments relating to the text,

Comment 1: Experimental Validation of Identified TFs: The identification of the 8 key transcription factors relies solely on computational analysis. To confirm their differential expression and biological relevance, the mRNA and protein levels of these TFs must be experimentally validated. This should involve direct comparison between primary AML patient samples and healthy donor samples (e.g., via qRT-PCR and Western blotting/immunohistochemistry).

Response: We sincerely appreciate the respected reviewer for his/her insightful comment which actually led to improvement of our paper.

This study is an in silico investigation that uses computational modeling to predict outcomes based on existing experimental data that are available in bioinformatics databases. These predictions are intended to guide the design of future laboratory experiments and to refine or expand previous studies. While experimental validation is indeed critical for confirming the predictions made, such validations fall beyond the scope of the current computational work. However, in line with your valuable feedback, we have incorporated a suggestion at the end of the revised manuscript to encourage further experimental studies to complement and extend the findings of this in silico work.

Comment 2: Functional Validation of Drug Resistance Role: The proposed link between these TFs and drug resistance is derived computationally. Functional experiments are essential to support this claim. Perform in vitro and/or in vivo assays (e.g., knockdown/overexpression followed by drug sensitivity testing in AML cell lines or primary cells) to demonstrate a causal role for these TFs in mediating drug resistance or sensitivity.

Response: we sincerely appreciate your insightful comment regarding the need for experimental support. As mentioned in response to previous comment, this study is an in silico investigation, and its primary objective is to generate predictive models based on existing experimental data to guide and optimize future laboratory experiments. Hence, the experimental investigations fall beyond the scope of the current computational work. To address your valuable suggestion, we have emphasized in the revised manuscript the potential experimental approaches that could be pursued to test our predictions, thereby fostering collaboration between computational and experimental research efforts.

Minor concerns;

1) Clarify Data Analysis Workflow: Given the multi-step bioinformatic analysis involving various filters, databases, and tools, include a clear data analysis flow diagram or schematic in the manuscript. This is crucial for transparency and reproducibility.

Response: This is a good suggestion. We added the overall Workflow of current study at the end of Method section (Figure 1). Accordingly, the numbering of figs was changed.

2. Correct Figure 7 Issues:

Labels Missing: Add the missing labels "a" and "b" to the panels in Figure 7.

Legend Discrepancy:Ensure the legend description for Figure 7a and 7b accurately reflects what is shown in the respective panels (currently described oppositely, lines 233-234).

Response: Thank you very much for your careful attention. You are absolutely right. The mentioned points regarding Figure 7 (Figure 8 in the revised version) have been corrected.

3. Correct Table 2: Fix the misspelling of "summary statistics" in Table 2.

Response: So many thanks. It has been corrected.

4. Correct Table 4 Legend: The legend for Table 4 (line 269) states there are 12 genes, but the table lists 24 genes (AML-up and AML-down). Update the legend to accurately reflect the total number of genes presented (24).

Response: Thank you very much for your insightful attention. You are right. It was corrected.

5. Clarify Table References: There is confusion regarding Table 5:

* Line 242 ,249 mentioned Table 5.

* Confirm and correct all table references. If Table 5 is mentioned in the text, it must exist and be numbered correctly. If it is a typo, correct all instances to refer to the appropriate table (likely Table 4).

Response: Thank you very much for your important comment. This mistake is due to transferring of some tables into the supplementary files. The Table 5 is indeed Table 3. Thanks to your nice comment this issue has been amended.

6. Revise Figure 9:

Resolution: Provide a high-resolution version of Figure 9, as the current version is illegible.

Legend Accuracy: The current legend description (lines 291-295) does not match the content of Figure 9. Completely revise the legend to accurately describe the data and analysis presented in the figure.

Response: Thank you very much for your comment. I think that the original image changes during conversion and inclusion into the pdf file by journal server. This image (Fig 10 in the revised version of the manuscript) is the output of the server. We have tried to manipulate the image by re-writing the names of the genes and cancer types. We also completely changed the legend of the Figure 10 according to your insightful; comment.

7. Clarify Figure 10: The text (line 309) and Figure 10a list only 7 genes. Confirm whether FEN1 is missing from both the text description and Figure 10a. If FEN1 is part of the analysis, it must be added to both places. If not, ensure the text and figure consistently reference the correct number (7) and list of genes.

Response: Thank you very much for your comment, which reflects a thorough and careful reading of the paper. The reason for the absence of the FEN1 gene in the figure is that, based on the database criteria and an FDR of less than 0.05, this gene did not show significant resistance or sensitivity to any of the analyzed drugs. Overall, the concept and interpretation of the figures in this chart are based on the CTRP and GDSC databases, relying on the following two assumptions:

1) Positive correlation indicates a higher gene expression may lead to drug resistance.

2) Negative correlation indicates a higher gene expression may make drug sensitive

Considering your comment, we added an explanatory statement in the Figure legend (Figure 11 in the revised version of the manuscript).

Reviewer #2:

The paper presents multi-step bioinformatics study to identify prognostic genes in Acute Myeloid Leukemia (AML) and explore their relationship with drug sensitivity. The authors utilize public gene expression data (GEO-GSE9476) and performs various bioinformatic approaches including differential expression analysis, pathway and network analysis, survival analysis, and finally, drug sensitivity correlation. They identified 8-gene signature (3 upregulated, 5 downregulated) and this is a potentially valuable finding. The use of multiple databases (GEO, GEPIA2, BloodSpot, GDSC) for analysis and validation is also a strength of this paper.

Comment 1: Authors have identified MCM2 and MCM3 as genes that are downregulated in AML patients compared to healthy controls. However, when discussing the drug sensitivity results from the CTRP database (Figure 10b), the text states to "MCM2, MCM3 (upregulated in AML patients)". Please clarify.

Response: Thank you very much for your careful attention. You are absolutely right. It was corrected.

Comment 2: The authors have marked the Ethics Statement as "N/A". This is inappropriate for a study that analyzes human patient and donor data, even if obtained from a public repository. The authors must correct this section to state that the data were obtained from a publicly available source (GEO-GSE9476) and that the original study that generated the data (Stirewalt et al.) received the necessary ethical approvals and patient consent, citing that study.

Response: Thank you very much for your nice comment. Thanks to your experience, it will be amended during submission process.

Comment 3: The process for selecting the final list of transcription factors in Table 1 is not detailed. The authors mention using "the statistical significance of the intersections and the repeatability of TFs across various databases". Please provide specific p-value thresholds and a clear definition of "repeatability."

Response: Thank you very much for your important comment. The missing information was added into the material and method section.

Comment 4: The criteria for selecting the hub genes listed in Table 3 are not explicitly stated. It is unclear if they are the top N genes by degree or another centrality metric. This should be clarified.

Response: So many thanks for your insightful comment. In line with your comment, we added more explanatory statements in the revised version of the manuscript (Section 3.4.1. Hub Genes and Significant Modules of the PPI Network)

Comment 5: The manuscript states that the 8 identified genes were associated with a "strong prognosis, suggesting high hazard ratios (HR) for AML". A high Hazard Ratio (HR > 1) typically signifies a worse prognosis (i.e., higher risk of a negative event, like death). The term "strong prognosis" is unclear. Please report whether high expression of a gene correlates with better or worse survival.

Response: Thank you very much for your suggestion. You are right. The current statement is confusing for readers. We applied some changes in the corresponding paragraph. The Legend of the Figure 10 has also changed according to your comment.

Comment 6: The connection to "epigenetics and lifestyle" is mentioned in the abstract, discussion, and conclusion as a potential factor influencing gene expression and drug response. This is not directly supported by any data in the manuscript. Stating them multiple times without support of data weakens the manuscript.

Response: Thank you very much for your insightful comment. Indeed, our aim was to extend the results of recent modeling studies—specifically those concerning the influence of expression patterns of certain genes on AML—toward a more generalized biological reality to provide a biologically meaningful interpretation of these findings. To this end, we discussed how gene expression patterns may be influenced by environmental factors. However, as you correctly pointed out, such a conclusion cannot be drawn directly or with absolute certainty. Therefore, in response to your comment, we have softened the wording of

Attachment Submitted filename: Rebuttal Letter.docx

# Peer review: Decision Letter 2

**DOI:** 10.1371/journal.pone.0352167.r005

**Authors:** Purnima Singh, Mohammad H. Ghazimoradi, Johnson Rajasingh

6 Mar 2026

PONE-D-25-22273R2

A systems biology approach to find representative genes in Acute Myeloid Leukemia

PLOS One

Dear Dr. Kalifeh,

Thank you for submitting your manuscript to PLOS ONE. After careful consideration, we feel that it has merit but does not fully meet PLOS ONE’s publication criteria as it currently stands. Therefore, we invite you to submit a revised version of the manuscript that addresses the points raised during the review process.

==============================

The manuscript can be accepted after verifying the following minor corrections:

·    In the *Methods*  section, ensure a clear distinction between the microarray platform (GEO-GPL96) and the dataset (GEO-GSE9476).

·    In the *Methods*  section, include explicit details regarding the statistical thresholds (p-values) and the criteria for “repeatability” used in transcription factor selection.

·    Verify the consistency in the expression status of **MCM2**  and **MCM3**  between the main text and the drug sensitivity figure, and correct if necessary for accuracy.

·    Clarify the **hub gene selection process**  in Section 3.4.1 by specifying the **centrality metrics**  used.

·    Confirm that the **interpretation of survival results**  has been revised to clearly indicate whether higher gene expression correlates with better or worse prognosis.

==============================

Please submit your revised manuscript by April 18, 2026. If you will need more time than this to complete your revisions, please reply to this message or contact the journal office at plosone@plos.org. When you're ready to submit your revision, log on to https://www.editorialmanager.com/pone/ and select the 'Submissions Needing Revision' folder to locate your manuscript file.

Please include the following items when submitting your revised manuscript:

- A letter that responds to each point raised by the academic editor and reviewer(s). You should upload this letter as a separate file labeled 'Response to Reviewers'.
- A marked-up copy of your manuscript that highlights changes made to the original version. You should upload this as a separate file labeled 'Revised Manuscript with Track Changes'.
- An unmarked version of your revised paper without tracked changes. You should upload this as a separate file labeled 'Manuscript'.

-->If you would like to make changes to your financial disclosure, please include your updated statement in your cover letter. Guidelines for resubmitting your figure files are available below the reviewer comments at the end of this letter.

If applicable, we recommend that you deposit your laboratory protocols in protocols.io to enhance the reproducibility of your results. Protocols.io assigns your protocol its own identifier (DOI) so that it can be cited independently in the future. For instructions see: https://journals.plos.org/plosone/s/submission-guidelines#loc-laboratory-protocols. Additionally, PLOS ONE offers an option for publishing peer-reviewed Lab Protocol articles, which describe protocols hosted on protocols.io. Read more information on sharing protocols at https://plos.org/protocols?utm_medium=editorial-email&utm_source=authorletters&utm_campaign=protocols.

We look forward to receiving your revised manuscript.

Kind regards,

Johnson Rajasingh, Ph.D, HCLD

Academic Editor

PLOS One

Journal Requirements:

If the reviewer comments include a recommendation to cite specific previously published works, please review and evaluate these publications to determine whether they are relevant and should be cited. There is no requirement to cite these works unless the editor has indicated otherwise.

Please review your reference list to ensure that it is complete and correct. If you have cited papers that have been retracted, please include the rationale for doing so in the manuscript text, or remove these references and replace them with relevant current references. Any changes to the reference list should be mentioned in the rebuttal letter that accompanies your revised manuscript. If you need to cite a retracted article, indicate the article’s retracted status in the References list and also include a citation and full reference for the retraction notice.

Additional Editor Comments (if provided):

[Note: HTML markup is below. Please do not edit.]

Reviewers' comments:

Reviewer's Responses to Questions

**Comments to the Author**

1. If the authors have adequately addressed your comments raised in a previous round of review and you feel that this manuscript is now acceptable for publication, you may indicate that here to bypass the “Comments to the Author” section, enter your conflict of interest statement in the “Confidential to Editor” section, and submit your "Accept" recommendation.

Reviewer #3: All comments have been addressed

**********

2. Is the manuscript technically sound, and do the data support the conclusions?

The manuscript must describe a technically sound piece of scientific research with data that supports the conclusions. Experiments must have been conducted rigorously, with appropriate controls, replication, and sample sizes. The conclusions must be drawn appropriately based on the data presented.

Reviewer #3: Yes

**********

3. Has the statistical analysis been performed appropriately and rigorously?

Reviewer #3: Yes

**********

4. Have the authors made all data underlying the findings in their manuscript fully available?

The PLOS Data policy requires authors to make all data underlying the findings described in their manuscript fully available without restriction, with rare exception (please refer to the Data Availability Statement in the manuscript PDF file). The data should be provided as part of the manuscript or its supporting information, or deposited to a public repository. For example, in addition to summary statistics, the data points behind means, medians and variance measures should be available. If there are restrictions on publicly sharing data—e.g. participant privacy or use of data from a third party—those must be specified.

Reviewer #3: Yes

**********

5. Is the manuscript presented in an intelligible fashion and written in standard English?

PLOS ONE does not copyedit accepted manuscripts, so the language in submitted articles must be clear, correct, and unambiguous. Any typographical or grammatical errors should be corrected at revision, so please note any specific errors here.

Reviewer #3: Yes

**********

6. Review Comments to the Author

Please use the space provided to explain your answers to the questions above. You may also include additional comments for the author, including concerns about dual publication, research ethics, or publication ethics. (Please upload your review as an attachment if it exceeds 20,000 characters)

Reviewer #3: (No Response)

**********

7. PLOS authors have the option to publish the peer review history of their article (what does this mean?). If published, this will include your full peer review and any attached files.

If you choose “no”, your identity will remain anonymous but your review may still be made public.

**Do you want your identity to be public for this peer review?**  For information about this choice, including consent withdrawal, please see our Privacy Policy.

Reviewer #3: No

**********

[NOTE: If reviewer comments were submitted as an attachment file, they will be attached to this email and accessible via the submission site. Please log into your account, locate the manuscript record, and check for the action link "View Attachments". If this link does not appear, there are no attachment files.]

To ensure your figures meet our technical requirements, please review our figure guidelines: https://journals.plos.org/plosone/s/figures

You may also use PLOS’s free figure tool, NAAS, to help you prepare publication quality figures: https://journals.plos.org/plosone/s/figures#loc-tools-for-figure-preparation.

NAAS will assess whether your figures meet our technical requirements by comparing each figure against our figure specifications.

Attachment Submitted filename: comments.docx

# Author comment: Author response to Decision Letter 3

**DOI:** 10.1371/journal.pone.0352167.r006

15 Apr 2026

Dear Editor

PLOS ONE

Manuscript ID: PONE-D-25-22273R2

Title: A systems biology approach to find representative genes in Acute Myeloid Leukemia

We sincerely appreciate the time and effort that you and the respected reviewer have dedicated to thoroughly evaluating our manuscript. We have carefully considered each point raised, and have addressed them in detail within manuscript. All changes in the final version of the manuscript were highlighted.

With kindest regards

Khosrow Khalifeh

________________________________________

comment 1:

The manuscript can be accepted after verifying the following minor corrections:

1) In the Methods section, ensure a clear distinction between the microarray platform (GEO-GPL96) and the dataset (GEO-GSE9476).

Response:

We thank the respected Editor for highlighting the need for clarity regarding the use of GEO-GPL96 and GEO-GSE9476 in our study. More explanation was added in the main text of the manuscript. (Pages 4 and 5)

comment 2: In the Methods section, include explicit details regarding the statistical thresholds (p-values) and the criteria for “repeatability” used in transcription factor selection.

Response: Thank you very much for your suggestion. The details of statistical criteria were added into the manuscript. (Page 7)

comment 3: Verify the consistency in the expression status of MCM2 and MCM3 between the main text and the drug sensitivity figure, and correct if necessary for accuracy.

Response: So many thanks for your comment. We added more statements to clarify our explanations. (Page 17)

comment 4: Clarify the hub gene selection process in Section 3.4.1 by specifying the centrality metrics used.

Response: Thank you very much for your suggestion. More explanatory statements were added into the revised version of manuscript. (Page 12)

comment 5: Confirm that the interpretation of survival results has been revised to clearly indicate whether higher gene expression correlates with better or worse prognosis.

Response: Thank you very much for your comment. We clarified our conclusion by adding more statements into the manuscript. (Pages 17 and 18)

Attachment Submitted filename: responses_to_reviewers_auresp_3.docx

# Peer review: Decision Letter 3

**DOI:** 10.1371/journal.pone.0352167.r007

**Authors:** Purnima Singh, Mohammad H. Ghazimoradi, Johnson Rajasingh, Mohamed Abdelkarim

7 Jun 2026

A systems biology approach to find representative genes in Acute Myeloid Leukemia

PONE-D-25-22273R3

Dear Dr.

We’re pleased to inform you that your manuscript has been judged scientifically suitable for publication and will be formally accepted for publication once it meets all outstanding technical requirements.

Within one week, you’ll receive an e-mail detailing the required amendments. When these have been addressed, you’ll receive a formal acceptance letter and your manuscript will be scheduled for publication.

An invoice will be generated when your article is formally accepted. Please note, if your institution has a publishing partnership with PLOS and your article meets the relevant criteria, all or part of your publication costs will be covered. Please make sure your user information is up-to-date by logging into Editorial Manager at Editorial Manager® and clicking the ‘Update My Information' link at the top of the page. For questions related to billing, please contact billing support.

If your institution or institutions have a press office, please notify them about your upcoming paper to help maximize its impact. If they’ll be preparing press materials, please inform our press team as soon as possible -- no later than 48 hours after receiving the formal acceptance. Your manuscript will remain under strict press embargo until 2 pm Eastern Time on the date of publication. For more information, please contact onepress@plos.org.

Kind regards,

Mohamed Abdelkarim

Academic Editor

PLOS One

Additional Editor Comments (optional):

Reviewers' comments:

Reviewer's Responses to Questions

**Comments to the Author**

1. If the authors have adequately addressed your comments raised in a previous round of review and you feel that this manuscript is now acceptable for publication, you may indicate that here to bypass the “Comments to the Author” section, enter your conflict of interest statement in the “Confidential to Editor” section, and submit your "Accept" recommendation.

Reviewer #3: All comments have been addressed

**********

2. Is the manuscript technically sound, and do the data support the conclusions?

The manuscript must describe a technically sound piece of scientific research with data that supports the conclusions. Experiments must have been conducted rigorously, with appropriate controls, replication, and sample sizes. The conclusions must be drawn appropriately based on the data presented.

Reviewer #3: Yes

**********

3. Has the statistical analysis been performed appropriately and rigorously?

Reviewer #3: Yes

**********

4. Have the authors made all data underlying the findings in their manuscript fully available?

The PLOS Data policy requires authors to make all data underlying the findings described in their manuscript fully available without restriction, with rare exception (please refer to the Data Availability Statement in the manuscript PDF file). The data should be provided as part of the manuscript or its supporting information, or deposited to a public repository. For example, in addition to summary statistics, the data points behind means, medians and variance measures should be available. If there are restrictions on publicly sharing data—e.g. participant privacy or use of data from a third party—those must be specified.

Reviewer #3: Yes

**********

5. Is the manuscript presented in an intelligible fashion and written in standard English?

PLOS ONE does not copyedit accepted manuscripts, so the language in submitted articles must be clear, correct, and unambiguous. Any typographical or grammatical errors should be corrected at revision, so please note any specific errors here.

Reviewer #3: Yes

**********

6. Review Comments to the Author

Please use the space provided to explain your answers to the questions above. You may also include additional comments for the author, including concerns about dual publication, research ethics, or publication ethics. (Please upload your review as an attachment if it exceeds 20,000 characters)

Reviewer #3: The suggested corrections have been adequately addressed, and the manuscript can be accepted in its current form.

**********

7. PLOS authors have the option to publish the peer review history of their article (what does this mean?). If published, this will include your full peer review and any attached files.

If you choose “no”, your identity will remain anonymous but your review may still be made public.

**Do you want your identity to be public for this peer review?**  For information about this choice, including consent withdrawal, please see our Privacy Policy.

Reviewer #3: **Yes:**  Seetharaman Jayaraman

**********

# Editor report: Acceptance letter

**DOI:** 10.1371/journal.pone.0352167.r008

**Authors:** Purnima Singh, Mohammad H. Ghazimoradi, Johnson Rajasingh, Mohamed Abdelkarim

PONE-D-25-22273R3

PLOS One

Dear Dr. Khalifeh,

I'm pleased to inform you that your manuscript has been deemed suitable for publication in PLOS One. Congratulations! Your manuscript is now being handed over to our production team.

At this stage, our production department will prepare your paper for publication. This includes ensuring the following:

* All references, tables, and figures are properly cited

* All relevant supporting information is included in the manuscript submission,

* There are no issues that prevent the paper from being properly typeset

You will receive further instructions from the production team, including instructions on how to review your proof when it is ready. Please keep in mind that we are working through a large volume of accepted articles, so please give us a few days to review your paper and let you know the next and final steps.

Lastly, if your institution or institutions have a press office, please let them know about your upcoming paper now to help maximize its impact. If they'll be preparing press materials, please inform our press team within the next 48 hours. Your manuscript will remain under strict press embargo until 2 pm Eastern Time on the date of publication. For more information, please contact onepress@plos.org.

You will receive an invoice from PLOS for your publication fee after your manuscript has reached the completed accept phase. If you receive an email requesting payment before acceptance or for any other service, this may be a phishing scheme. Learn how to identify phishing emails and protect your accounts at https://explore.plos.org/phishing.

If we can help with anything else, please email us at customercare@plos.org.

Thank you for submitting your work to PLOS ONE and supporting open access.

Kind regards,

PLOS ONE Editorial Office Staff

on behalf of

Dr. Mohamed Abdelkarim

Academic Editor

PLOS One
